# Enhanced Stability in Zero‐Excess Li‐Metal Batteries via Prelithiated Carbon Nanofiber Interlayers

**DOI:** 10.1002/advs.75690

**Published:** 2026-05-14

**Authors:** Sandro Schöner, Marius Ast, Vera Michaela Barysch, Rebecca Erkes, Jule Meier‐Merziger, Pengfei Cao, Joachim Mayer, Josef Granwehr, Fabian Jeschull, Hermann Tempel, Shicheng Yu, Rüdiger‐A. Eichel

**Affiliations:** ^1^ Institute of Energy Technologies–Fundamental Electrochemistry (IET‐1) Forschungszentrum Jülich Jülich Germany; ^2^ Institute of Physical Chemistry–Material and Processes of Electrochemical Energy Storage and Conversion RWTH Aachen University Aachen Germany; ^3^ Institute for Applied Materials (IAM) Karlsruhe Institute of Technology (KIT) Eggenstein‐Leopoldshafen Germany; ^4^ Institute of Technical and Macromolecular Chemistry RWTH Aachen University Aachen Germany; ^5^ Ernst Ruska‐Centre for Microscopy and Spectroscopy with Electrons Forschungszentrum Jülich Jülich Germany; ^6^ Central Facility for Electron Microscopy (GFE) RWTH Aachen University Aachen Germany; ^7^ Faculty of Mechanical Engineering RWTH Aachen University Aachen Germany

**Keywords:** 3D interlayer, artificial SEI, carbon nanofiber, Li deposition, prelithiation, zero‐excess Li metal battery

## Abstract

*Zero‐excess* lithium metal batteries (ZELMBs), here referring to the absence of Li‐foil on the anode side rather than the strict absence of additional lithium inventory, are considered a promising approach to increase energy density, but their performance is often limited by dendritic lithium growth and low Coulombic efficiency (CE). Here, we explore a chemically prelithiated carbon nanofiber (CNF) interlayer as a lithiophilic host to mitigate these challenges. Advanced characterization techniques, including solid‐state NMR, Raman spectroscopy, and depth‐profiling XPS, suggest that the prelithiation process leads to the formation of an inorganic‐rich interphase containing LiOH, Li_2_O, and lithium‐containing organic species. This interphase is associated with reduced nucleation overpotential and more uniform lithium deposition, helping to suppress dendritic growth. Furthermore, prelithiation introduces additional lithium inventory into the CNF interlayer, a fraction of which may compensate for initial lithium loss during early cycling. As a result, CNF_Prelith_ paired with LiFePO_4_ cathodes demonstrates improved cycling stability, with CE exceeding 99.95% and good capacity retention at elevated C‐rates (up to 4 C). Notably, stable performance is achieved without extended formation protocols in full cell configurations. These findings indicate that prelithiated interlayers can be a useful strategy for stabilizing Li‐foil‐free lithium metal cells under limited‐lithium conditions.

## Graphical abstract

Carbon nanofibers carbonized at 700°C show Li metal deposition predominantly on the side facing the Cu current collector. During delithiation, Li agglomerates remain as inactive lithium on the fiber surface. This behavior can be changed by a modified *n*‐Buli prelithiation without a washing step, suppressing dead Li formation and therefore enabling stable cycling.

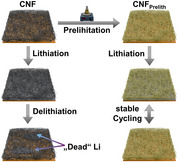

## 1 Introduction

The pursuit of higher‐energy rechargeable batteries has intensified interest in replacing conventional graphite anodes with lithium metal, which offers the highest known theoretical capacity (3860 mAh g^−1^) and the low electrochemical potential (−3.04 V vs. Li/Li^+^) [1]. However, despite these remarkable properties, traditional Li‐metal anodes introduce substantial excess lithium metal to compensate for severe irreversible losses, side reactions, and unstable cycling behavior [2, 3]. *Zero‐excess* lithium metal batteries (ZELMBs) have emerged as an alternative configuration in which no Li metal foil is preloaded at the anode [4]. This conceptual transition dramatically increases the achievable gravimetric and volumetric energy densities of full cells by eliminating inactive materials and conventional anode components such as lithium metal or graphite [5, 6]. Furthermore, the absence of preloaded lithium simplifies manufacturing and reduces cost, positioning ZELMBs as a promising pathway toward next‐generation high‐energy systems [7, 8]. Yet despite these advantages, the low stability of lithium plating and stripping under *zero‐excess* lithium metal conditions remains the primary obstacle to practical implementation [9].

Several intrinsic bottlenecks fundamentally limit the electrochemical stability of *zero‐excess* lithium metal configurations. Because the initial deposition of lithium occurs directly on a bare current collector, typically copper, the interfacial energy landscape is highly unfavorable and promotes inhomogeneous nucleation [10, 11]. This leads to uncontrolled dendritic growth, surface‐area formation, and dead lithium accumulation from the very first cycle, resulting in rapid capacity decay and Coulombic efficiency losses [12]. In addition, the absence of excess lithium metal amplifies irreversible side reactions, including continuous solid‐electrolyte interphase (SEI) reconstruction, electrolyte decomposition, and progressive loss of lithium inventory [13, 14]. The SEI layer formed on a pristine current collector is often chemically unstable, mechanically fragile, and incapable of suppressing dendritic propagation under high current densities [15]. Furthermore, the dynamic volume fluctuations associated with lithium deposition exacerbate interfacial cracking and expose fresh lithium to the electrolyte, thereby accelerating degradation processes [16]. Collectively, these issues result in narrow operational windows, limited areal capacities, and insufficient cycle life that fail to meet the requirements of practical battery applications [17].

To overcome these challenges, extensive research has focused on engineering strategies that stabilize lithium deposition and improve SEI robustness in anode‐free systems. One widely explored approach involves modifying the current collector surface using textured or chemically functionalized substrates to lower the nucleation overpotential and promote uniform lithium plating [18, 19, 20]. Artificial SEI layers, constructed through chemical, electrochemical, or vapor‐phase routes, have also demonstrated the ability to enhance interphase stability and suppress dendritic growth [21]. Electrolyte optimization, such as the use of fluorinated solvents, high‐concentration electrolytes, or localized high‐concentration electrolytes, can further mitigate side reactions and promote the formation of LiF‐rich SEI structures with improved mechanical integrity [22]. Another promising direction lies in the introduction of lithiophilic coatings or interlayers, which guide lithium‐ion flux and act as hosts for controlled nucleation, thereby reducing the likelihood of mossy or dendritic morphologies [23]. Despite these advances, truly reliable *zero‐excess* lithium metal operation remains elusive, underscoring the need to design multifunctional interlayers that combine structural guidance, chemical stabilization, and compatibility with practical manufacturing processes [24, 25].

Among these interfacial engineering strategies, three‐dimensional (3D) carbon‐based interlayers, especially carbon nanofiber (CNF) networks, have attracted significant attention due to their high surface area, lightweight structure, mechanical robustness, and excellent electronic conductivity [26, 27]. These CNF structures can distribute the effective current density, accommodate lithium within porous domains, and provide continuous conductive pathways during cycling [28]. Moreover, their tunable surface chemistry enables the incorporation of functional groups that can enhance lithiophilicity [29, 30]. However, despite these advantages, CNF interlayers face several persistent challenges when operated under *zero‐excess* lithium metal conditions. First, pristine carbon surfaces generally exhibit limited intrinsic affinity for lithium, resulting in delayed or heterogeneous nucleation [31]. Second, electrolyte decomposition on high‐surface‐area carbon can lead to unstable or excessively thick SEI layers that consume active lithium, which is particularly detrimental in *zero‐excess* lithium metal configurations where the lithium inventory is strictly limited [32, 33]. Third, uncontrolled lithiation of carbon, especially in highly defective or oxygen‐rich CNFs, may compete with metallic lithium deposition, reducing CE and shifting the cell away from reversible metal‐plating behavior [34]. These drawbacks illustrate that unmodified CNF interlayers, while structurally promising, require targeted chemical engineering to fully satisfy the stringent constraints of ZELMBs.

Despite recent efforts to improve interfacial stability using artificial SEI layers, 3D hosts, and lithiophilic coatings, these strategies often rely on excess lithium or cycling protocols that mask intrinsic instability. Consequently, regulating lithium nucleation and sustaining reversible lithium transport in *zero‐excess* lithium metal configurations remain critical challenges, particularly as the interphase chemistry of carbon scaffolds evolves dynamically. A rational design that simultaneously addresses the host's chemical reactivity, the SEI's mechanical resilience, and the homogeneity of Li^+^ flux is still lacking.

Here, we introduce a chemically prelithiated carbon nanofiber (CNF_Prelith_) interlayer designed to stabilize Li nucleation from the very first cycle. Distinct from electrolyte engineering, this approach leverages controlled reactions between *n*‐butyllithium (*n*‐BuLi) and surface functional groups to construct a Li‐containing interface prior to cell assembly. This strategy enables the decoupling of intrinsic surface chemistry from electrochemically induced instability, allowing a systematic investigation of how prelithiated surfaces govern structural integrity and SEI formation pathways. In this study, the term “*zero‐excess* Li metal” is used operationally to denote the absence of Li metal foil on the anode side. It does not imply the complete absence of additional lithium inventory, because the prelithiated CNF introduces extra lithium into the system. By integrating multimodal characterization with electrochemical analysis, this work establishes a mechanistic understanding of prelithiation‐engineered interfaces and demonstrates their potential for practical, high‐energy‐density batteries under these conditions at the laboratory scale.

## 2 Results and Discussion

**FIGURE 1:**
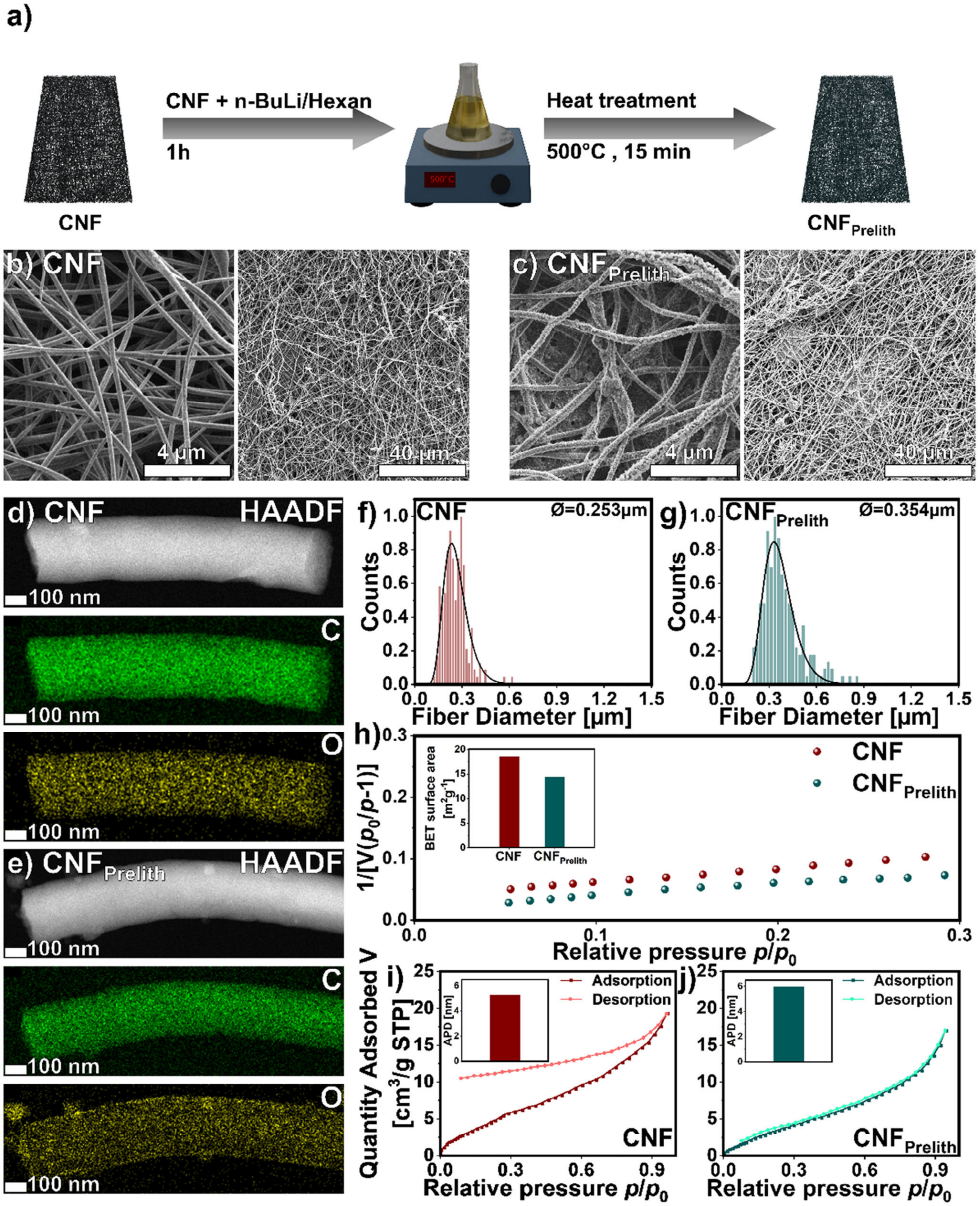
(a) Schematic illustration of the prelithiation process. (b) SEM images of CNF, (c) SEM images of CNF_Prelith_ (d) High‐Angle Annular Dark‐Field (HAADF) – STEM‐images and STEM‐EDX elemental mappings of C and O for CNF (e) HAADF‐STEM‐images and STEM‐EDX elemental mappings of C and O for CNF_Prelith_. Distribution of fiber diameters for (f) CNF and (g) CNF_Prelith._ (h) Ar adsorption isotherm of CNF and CNF_Prelith_ measured at 273 K and the calculated BET surface area. Ar adsorption‐desorption isotherms and the average pore diameter (APD) for (i) CNF and (j) CNF_Prelith_.

The structural and chemical properties of CNF and CNF_Prelith_ were systematically investigated to elucidate how prelithiation influences lithium nucleation, interfacial stability, and overall electrochemical performance. The pristine CNFs were synthesized via electrospinning, followed by carbonization at 700°C under argon atmosphere. These fibers were chemically prelithiated by immersion in 1 mL 2.5 M *n‐*BuLi hexane solution as illustrated in Figure 1a and subsequently annealed at 500°C for 15 min to remove excess solvent and to enable surface reactions induced by the decomposition of *n*‐BuLi. Scanning electron microscopy (SEM) images of pristine CNF (Figure 1b) reveal a disordered, porous 3D network, indicative of a high specific surface area and abundant accessible pathways for lithium ions. In comparison, high‐resolution SEM images of CNF_Prelith_ (Figure 1c) display a surface layer with more granular features, which are notably absent in pristine CNFs. This morphological transformation suggests the formation of lithium‐containing surface species, potentially including Li_2_O, LiOH, lithium‐containing organic compounds, and possibly LiH‐related species as a result of the chemical decomposition of *n‐*BuLi. [35] Importantly, the underlying fiber network remains structurally intact, demonstrating that prelithiation modifies the surface while maintaining the bulk morphology, which is crucial for preserving electronic conductivity and mechanical robustness. Figure 1d,e illustrates scanning transmission electron microscopy (STEM) coupled with energy‐dispersive X‐ray spectroscopy (EDX) measurements. They include field‐of‐view images and elemental mapping for carbon (C), oxygen (O), and nitrogen (N) (Figure S1). Li cannot be detected using EDX techniques and is therefore not illustrated. Plan‐view STEM images of a fiber sample, shown in Figure S2, further confirm that the bulk morphology remains intact since no change can be observed in the elemental mapping of C, O, and N. However, analysis of the fiber diameter of CNF illustrated in Figure 1f,g, calculated from the SEM images shown in Figure 1b,c, reveals that the average fiber diameter increases from 0.253 µm ± 0.1 µm to 0.354 µm ± 0.1 µm with prelithiation, suggesting a reaction between the CNF and Li‐ions during prelithiation. A similar trend is observed with the Brunauer–Emmett–Teller (BET) measurements (Figure 1h), which show a reduction in the specific surface area from 18.6 to 14.4 m^2^g^−1^ (±1%) after prelithiation. This reduction can be attributed to partial pore coverage and surface coverage due to lithium‐containing component deposition during *n*‐BuLi decomposition on the surface.

Argon adsorption and desorption isotherms (Figure 1i,j) reveal notable differences in pore accessibility. Pristine CNFs exhibit a pronounced hysteresis between adsorption and desorption, suggesting that pores smaller than the measured average of 5.28 nm exist within the fiber, thereby hindering the desorption process. In contrast, CNF_Prelith_ shows nearly overlapping adsorption and desorption curves, indicating that prelithiation significantly reduces surface microporosity and results in an increased average pore of 5.98 nm. This pore filling or modification is consistent with the formation of a lithium‐rich surface layer, which not only stabilizes the fiber surface but may also enhance initial lithium nucleation, promote more uniform Li plating, and thereby reduce the risk of dendrite formation.

**FIGURE 2:**
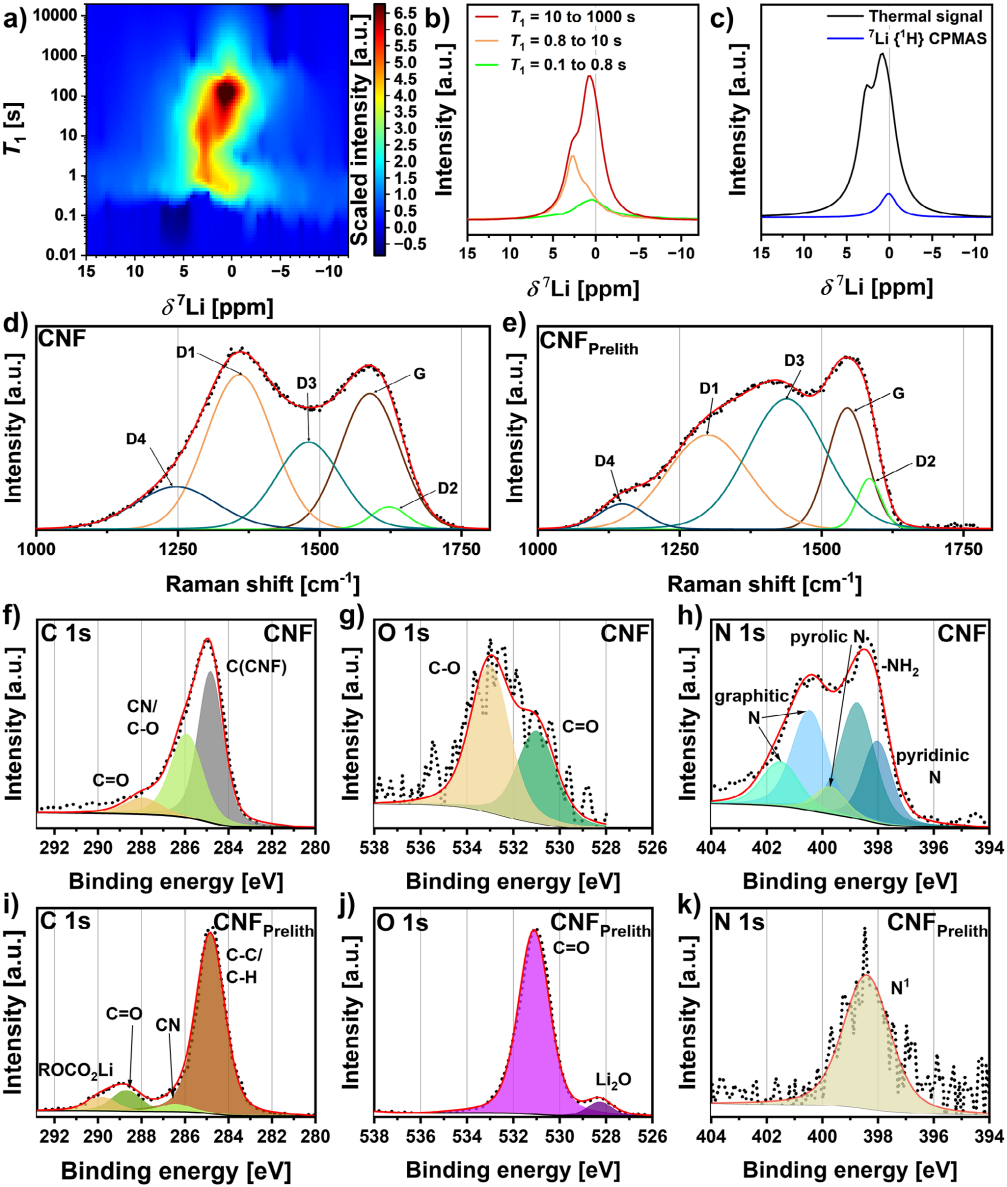
(a) Spin‐lattice relaxation time *T*_1_ distributions obtained via Inverse Laplace Transform of inversion‐recovery data from ^7^Li MAS NMR measurements at 18.8 T for the CNF_Prelith_ sample. The intensities were scaled by ‐sign(g)∙|g|^0.5^ to enhance smaller features. (b) Cumulative spectra from the ILT data for three *T*_1_ ranges (0.1–0.8 s, 0.8–10 s, and 10–1000 s). (c) ^7^Li{^1^H} CPMAS spectrum (blue) compared to the thermal MAS spectrum (black). A reference line at 0 ppm was added in (b) and (c) for orientation. (d) Raman spectra of CNF and (e) Raman spectra of CNF_Prelith_. Spectral parameters for the Raman fitting are listed in Table S1. (f) C 1s, (g) O 1s, and (h) N 1s XPS spectra of CNF, (i) C 1s, (j) O 1s, and (k) N 1s of CNF_Prelith_. The spectra for CNF are referenced to 398.0 eV for the pyridinic nitrogen, and the spectra of CNF_Prelith_ are referenced to 284.8 eV for the signal corresponding to C─C/C─H. All spectra are normalized, with the highest signal in each spectrum set to 1. Survey measurements are provided in Figure S3. Detailed measurement parameters are listed in Table S2. The fitting parameters are listed in Tables S3 and S4.

Solid‐state ^7^Li magic‐angle spinning (MAS) nuclear magnetic resonance (NMR) measurements were used to obtain additional details of the surface chemistry after prelithiation. The thermally polarized spectrum (Figure 2c) shows multiple resonances in the frequency range associated with Li in diamagnetic environments, which is known to cover a narrow chemical shift range [36]. Further line broadening caused by quadrupolar interactions that are not perfectly averaged out by MAS challenges a clear separation or even an unambiguous accounting of the number of contributing resonances, yet two maxima are apparent at around 0.7 ppm and 2.6 ppm. Resolving an independent property in a second dimension is a common strategy to facilitate better separation of overlapping resonances in NMR [37]. In Figure 2a the ^7^Li MAS NMR spectrum is resolved with respect to the longitudinal relaxation time *T*_1_ using the subsequent Inverse Laplace Transform (ILT) [38]. The resulting 2D map reveals at least three distinct lithium environments in CNF_Prelith_, distinguished by their chemical shifts and *T*_1_ values (Figure 2a), two of which overlap in the spectral dimension yet are clearly distinguishable by *T*_1_. ^7^Li *T*_1_ values are known to show pronounced contrast regarding the Li ion mobility. At room temperature, low mobility or immobile ^7^Li ions in diamagnetic, crystalline inorganic environments typically show *T*_1_ > 10 s, while for highly mobile ^7^Li often *T*_1_ < 1 s is observed. Since ^7^Li *T*_1_ is a very sensitive probe of changes in the environment, in Li‐ion conducting environments, considerable *T*_1_ distributions can be found [39]. For direct comparison, cumulative spectra are depicted for three *T*_1_ ranges (Figure 2b). For the *T*_1_ range of 10 to 1000 s, the most intense signal is centered at 0.7 ppm. Such high *T*_1_ values for ^7^Li indicate rigid, immobile or low mobility lithium species. In CNF_Prelith_, these immobile species represent the largest Li contribution. A second signal, centered at 2.6 ppm, dominates within the *T*_1_ range from 0.8 to 10 s. This chemical shift range has been reported for Li_2_O [40]. The broad *T*_1_ distribution of this signal indicates that the corresponding species can be found in the bulk below the surface, resulting in higher *T*_1_ values. In contrast, species located at oxygen‐related defect sites near the surface are more likely to participate in reactions during the *n*‐BuLi treatment, potentially resulting in the formation of lithium oxygen species similar to Li_2_O, resulting in shorter *T*_1_ values. For the *T*_1_ range of 0.1–0.8 s a weaker signal is observed, centered at 0.3 ppm, indicating either a more mobile environment or Li ions in immediate contact with electron‐conducting domains. In the context of Li in an SEI, more organic, soft species sometimes associated with an outer SEI layer may be causing such a signal. To investigate the proximity of protons, which would be present in such an environment, ^7^Li{^1^H} cross‐polarization (CP) MAS experiments were performed (Figure 2c). The ^7^Li{^1^H} CPMAS spectrum exhibits a single signal centered at 0.0 ppm. Comparing this result with the *T*_1_ relaxation data, the Li species in close proximity to the protons correspond to the short *T*_1_ regime (0.1–0.8 s). The short *T*_1_ in combination with proton contact indicates that this signal could be caused by an organic SEI layer or by an ionic surface species with lithium and proton contributions, such as LiOH [41]. Since the CP signal maximum differs by 0.3 ppm from the signal maximum for the short *T*_1_ range (0.1–0.8 s), this demonstrates that only one of the fast‐relaxing Li species exhibits sufficient dipolar coupling with protons to yield a CP signal.

Since the NMR results confirm the formation of an artificial SEI with immobile Li species as the main lithiation product, the corresponding structural evolution of the carbon host was analyzed using Raman spectroscopy (Figure 2c,d). To quantify the degree of disorder and structural composition, each spectrum was deconvoluted using a five‐band model. The spectra feature the characteristic G‐band (E_2g_ mode of sp^2^‐bonded carbon) typically near 1580 cm^−1^, the D1‐band (A_1g_ mode) around 1350 cm^−1^, arising from lattice defects, and the D2‐band associated with edge defects. Additionally, the D3 and D4 bands are assigned to amorphous carbon and heteroatom‐related surface structures, respectively.

Upon prelithiation, the G‐band exhibits a notable redshift from 1590 cm^−^^1^ in pristine CNF to 1547 cm^−^^1^ in CNF_Prelith._ This significant shift is consistent with lithium‐induced n‐doping, where electron transfer from Li to the carbon π* orbitals weakens the C─C bonds and induces lattice expansion, analogous to tensile strain [42, 43]. Quantitatively, the integrated area ratio A_D1_/A_G_ increased from 1.26 to 1.67, indicating disruption of the graphitic domains. More importantly, the degree of surface‐related disorder, evaluated by the relative contribution of the D3 and D4 bands (A_D3_ + A_D4_)/A_total_, increased from 0.33 to 0.46. This trend provides direct spectroscopic evidence for the formation of a disordered, chemically modified surface layer, which is critical for homogenizing Li nucleation.

XPS was employed to investigate the chemical transformations occurring on the surface of the CNFs during prelithiation with *n*‐BuLi. Pristine CNFs, synthesized from PAN precursors under controlled carbonization conditions, possess a variety of surface functionalities that act as reactive anchoring sites for *n*‐BuLi, providing the necessary chemical environment for subsequent surface reactions.

In the *C 1s* spectrum illustrated in Figure 2d of untreated CNFs, a dominant peak at 284.8 eV (CNF) is observed, accompanied by a secondary peak at 285.9 eV (C─O/C─N) and a minor component at 287.9 eV, which can be attributed to C═O. These oxygen‐containing components are likely introduced during the oxidative stabilization step and serve as nucleophilic and redox‐active centers that can readily participate in surface chemical reactions. The *O 1s* spectra presented in Figure 2f of pristine CNFs exhibit two distinct peaks at 531.0 eV and 533.0 eV, which can be attributed to C═O and C─O, likely corresponding to ketone, hydroxyl, or ether groups that are reactive with *n*‐BuLi. In addition, the *N 1s* spectrum in Figure 2g shows a complex profile with pyridinic nitrogen at 398.0 eV, ─NH_2_ groups at 398.7 eV, pyrrolic nitrogen at 399.7 eV, and graphitic nitrogen at 400.4 eV and 401.5 eV. These nitrogen species contribute to electronic conductivity and may also help stabilize lithium species at the fiber surface, thus enhancing the overall reactivity and surface chemistry of the CNFs [44].

Depth profiling, illustrated in Figure S4 reveals a nearly homogeneous distribution of C, O, and N throughout the fiber network. The intensities of *C 1s*, *O 1s*, and *N 1s* remain almost constant with increasing sputter depth, indicating that the surface chemistry is largely representative of the bulk material, which is consistent with the TEM results shown in Figure 1.

During the heating step of the prelithiation process, the reactive surface functionalities undergo a series of chemical reactions, including nucleophilic substitution and reduction, which collectively convert the fiber surface into an inorganic–organic hybrid SEI. In the *C 1s* spectrum of CNF_Prelith_, shown in Figure 2i, the sp^3^‐hybridized C─C/C─H peak at 284.8 eV dominates, while a weaker shoulder at 286.2 eV corresponds to residual C‐N environments originating from the bulk material. New peaks emerge at 288.8 eV and 289.9 eV, which are assigned to RCO_2_Li and ROCO_2_Li, marking the chemical transformation of the original C─O and C═O groups during prelithiation.

The *O 1s* spectrum of CNF_Prelith_, illustrated in Figure 2j, shows a main component at 531.0 eV, which can be attributed to components containing C═O and to LiOH. In addition, a new peak appears at 528.8 eV, assigned to Li_2_O, while the previous peak at 533.0 eV disappears. This confirms that the organic hydroxyl and ether functionalities are consumed during prelithiation to form new Li‐containing products. The formation of Li_2_O aligns with the expected reactivity of *n*‐BuLi toward surface oxygen groups. Collectively, these observations provide evidence for surface chemical reconstruction, in which the original reactive groups are systematically transformed into a stable lithium‐rich artificial SEI.

The attenuated *N 1s* signal in CNF_Prelith_, shown in Figure 2k, while still detectable, suggests that the SEI layer is sufficiently thin to allow detection of nitrogen originating from the underlying CNF substrate, consistent with the information depth of XPS (∼10 nm). Although only a single signal is observed in the *Li 1s* spectrum shown in Figure S5, this peak represents all previously identified lithium species. Because the binding energies of the individual components are very close in the *Li 1s* spectrum, deconvolution into separate signals was not performed. A quantitative analysis of the XPS data, summarized in Table S4, reveals a high lithium concentration of approximately 52 at.%, which cannot be accounted for solely by RCO_2_Li, ROCO_2_Li, and Li_2_O. At most 25.35 at.% of Li can be attributed to carbon‐containing species (and this value is likely overestimated, as C─C and C─H bonds in polymer chains do not contribute Li). After accounting for the oxygen associated with these carbon‐containing species, 10.97 at.% of O remains unassigned. Given that LiOH is reported near this binding‐energy range, the data are consistent with the presence of LiOH at the surface. This assignment is further supported by the XRD pattern shown in Figure S6 and by the NMR analysis. Despite these assignments, 15.5 at.% of Li remains unaccounted for. Since no metallic Li is present, which would be observed at lower binding energies, this residual Li is most plausibly assigned to a species containing only Li and H, such as LiH, which cannot be detected directly by XPS due to the absence of detectable hydrogen [45]. These rigid inorganic species (Li_2_O, LiOH, and LiH) in combination with the organic species RCO_2_Li and ROCO_2_Li form a mechanically robust artificial SEI with a high Li‐ion conductivity [46, 47, 48]. These interfacial properties may help reduce lithium loss associated with electrolyte decomposition and may improve tolerance to mechanical stress during Li deposition.

Depth profiling of CNF_Prelith_, shown in Figure S7, demonstrates that this transformation is highly localized at the CNF surface. The oxygen content is highest at the outermost surface (20.9 at.%) and decreases progressively to 6.5 at.% toward the fiber interior (∼2002 s sputter time). An increase in the Li_2_O content during depth profiling is commonly attributed to sputter‐induced damage. However, in combination with the NMR results presented above, which indicate that a significant fraction of Li_2_O is located below the surface, the observed species can be attributed to constituents of the lower layers of the artificial SEI. Nevertheless, a quantitative analysis should be avoided, as sputter‐induced damage may still contribute to an apparent increase in the Li_2_O content. The depth profile suggests a possible core‐shell‐like interphase architecture, with a lithium‐ and oxygen‐rich outer region and an electronically conductive carbon interior. If present, such an arrangement may contribute to the observed interfacial stability and electrochemical performance of CNF_Prelith_

To evaluate the practical effectiveness of CNF‐based interlayers in lithium metal batteries for lithium plating and stripping stability, electrochemical cells were assembled using either pristine CNF or CNF_Prelith_ as the interlayer against Li metal in a Cu/sample║electrolyte║Li‐metal configuration. The electrolyte consisted of 0.6 M LiDFOB and 0.6 M LiBF_4_ in FEC/DEC (1:2 by volume) [49]. These experiments were designed to assess the stability and efficiency of lithium plating and stripping, which are critical indicators of the suitability of interfacial materials in next‐generation *zero‐excess* lithium metal battery architectures.

**FIGURE 3:**
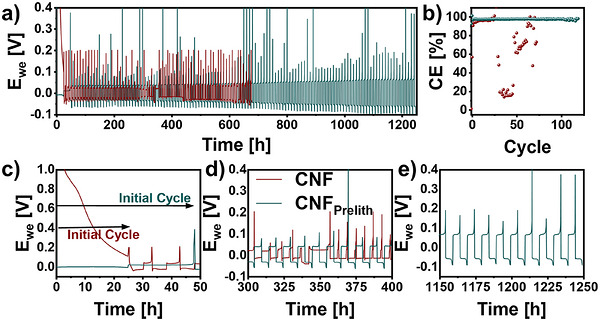
(a) Voltage – time profiles of CNF and CNF_Prelith_ during Li deposition/dissolution experiments at an areal capacity of 2.5 mA cm^−2^. (b) Corresponding coulombic efficiencies of the materials shown in (a). Magnified segments from (a) at different cycling stages: (c) initial cycle, (d) dendrite formation on CNF, and (e) stable cycling behavior of CNF_Prelith_.

To compare pristine and prelithiated CNF, the electrochemical protocol included an initial conditioning cycle, during which lithium was plated onto the interlayer at a low current density of 0.1 mA cm^−2^ for 25 h, followed by stripping until a cut‐off voltage of 0.4 V. In subsequent cycles, the plating current density was increased to 0.5 mA cm^−2^, while the deposition time was reduced to 5 h per half‐cycle, maintaining a constant areal capacity of 2.5 mA cm^−2^ per cycle. The corresponding voltage profiles and coulombic efficiencies (CEs) are presented in Figure 3.

For the pristine CNF interlayer, the initial plating behavior, revealed in the magnified view in Figure 3c, shows a very slow voltage decay over the 25‐h deposition period. The voltage gradually decreased and plateaued at 0.36 V, never reaching negative potential during the first cycle. This indicates that no substantial lithium metal deposition occurred on the CNF surface under these conditions. This observation is further confirmed by the calculated CE for the first cycle, which was below 1%. During the second cycle, the voltage profile displays negative potential during the plating half‐cycle, suggesting that lithium nucleation on the CNF framework had finally occurred. The CE correspondingly increased to 56.6 %, gradually improving in subsequent cycles to an average of 96.0 % after several stabilization cycles. The plateau at 0.36 V is characteristic of lithium intercalation into disordered carbon as well as the irreversible decomposition of electrolyte to form the SEI. Due to these side reactions, the applied current was consumed in irreversible processes, rather than forming metallic lithium.

These results indicate that pristine CNF exhibits relatively low initial lithiophilicity. The delayed nucleation and poor CE during the early cycles reflect the material's initial susceptibility to side reactions with defect sites and the formation of a native SEI. Extended cycling is required to establish a more favorable nucleation environment, which can be detrimental in *zero‐excess* lithium metal configurations, where early‐cycle irreversibility directly translates into capacity loss.

In contrast, the CNF_Prelith_ interlayer displayed markedly different behavior under the same conditions. Lithium nucleation occurred almost immediately, with the cell voltage becoming negative within 3 minutes of the first deposition process. This rapid lithiation is consistent with the presence of a preformed artificial SEI and a lithium‐rich surface layer, as previously characterized by XPS and Raman spectroscopy. The first cycle CE was 92.6 %, substantially higher than that of pristine CNF, which required three cycles to achieve comparable efficiency.

During subsequent cycles, CNF_Prelith_ maintained stable and efficient plating/stripping behavior. Coulombic efficiency steadily increased, reaching values exceeding 97.6%, which is higher than that of fully conditioned pristine CNF but achieved much earlier and with far greater consistency. This highlights the beneficial role of prelithiation in establishing an electrochemically favorable interface that enables homogeneous lithium nucleation, suppresses early‐cycle lithium loss, and mitigates dendrite formation. Moreover, no irreversible side reactions within the carbon framework were observed, as defect sites are preoccupied by lithium introduced during the prelithiation step, effectively acting as an internal lithium reservoir that buffers initial lithium depletion.

Magnified intervals at later points during cycling (Figure 3d,e) further underscore the impact of prelithiation. The pristine CNF cell, although eventually reaching high CE after extended cycling, started to exhibit voltage fluctuations after 300 h, indicative of micro‐short circuits caused by dendritic penetration and the continuous accumulation of electrically isolated lithium. After approximately 350 h the cell with the unmodified CNF as an interlayer failed due to Li dendrite formation. Even after 1250 h of continuous operation, the CE remains high and stable with minimal fluctuation, consistent with the formation of a mechanically robust and chemically resilient solid electrolyte interphase (SEI). This stability is further associated with prelithiation‐induced lithium incorporation within the host structure, which modulates the available lithium inventory and helps sustain interfacial equilibrium during extended cycling. In contrast, the bare Cu current collector tested under the same conditions, as shown in Figure S8, exhibits more rapid degradation, with cell failure occurring after 225 h, despite an initially higher Coulombic efficiency of 95.3%.

Further insights into suitability for fast charge–discharge operation were obtained from critical current tests (Figures S9 and S10) at capacities of 1.25 mA cm^−2^ and 2.5 mA cm^−2^, respectively. For these tests, the current density was increased stepwise every five cycles after initial formation. The pristine CNF interlayer demonstrated good critical current stability up to 3.5 mA cm^−2^ at 1.25 mA cm^−2^, attributed to its high surface area, which enables more even current distribution. However, at the higher capacity of 2.5 mA cm^−2^, critical current stability decreased to ∼1.0 mA cm^−2^, consistent with reduced interlayer stability under higher stress.

In contrast, cells with CNF_Prelith_ maintained notable critical current stability regardless of applied capacity, remaining stable up to 6.0 mA cm^−2^. This performance aligns with previous cycling results and highlights the potential of CNF_Prelith_ interlayers for fast‐charging ZELMBs, combining high initial CE with great stability and reversibility for the plating/stripping process of Li‐metal.

**FIGURE 4:**
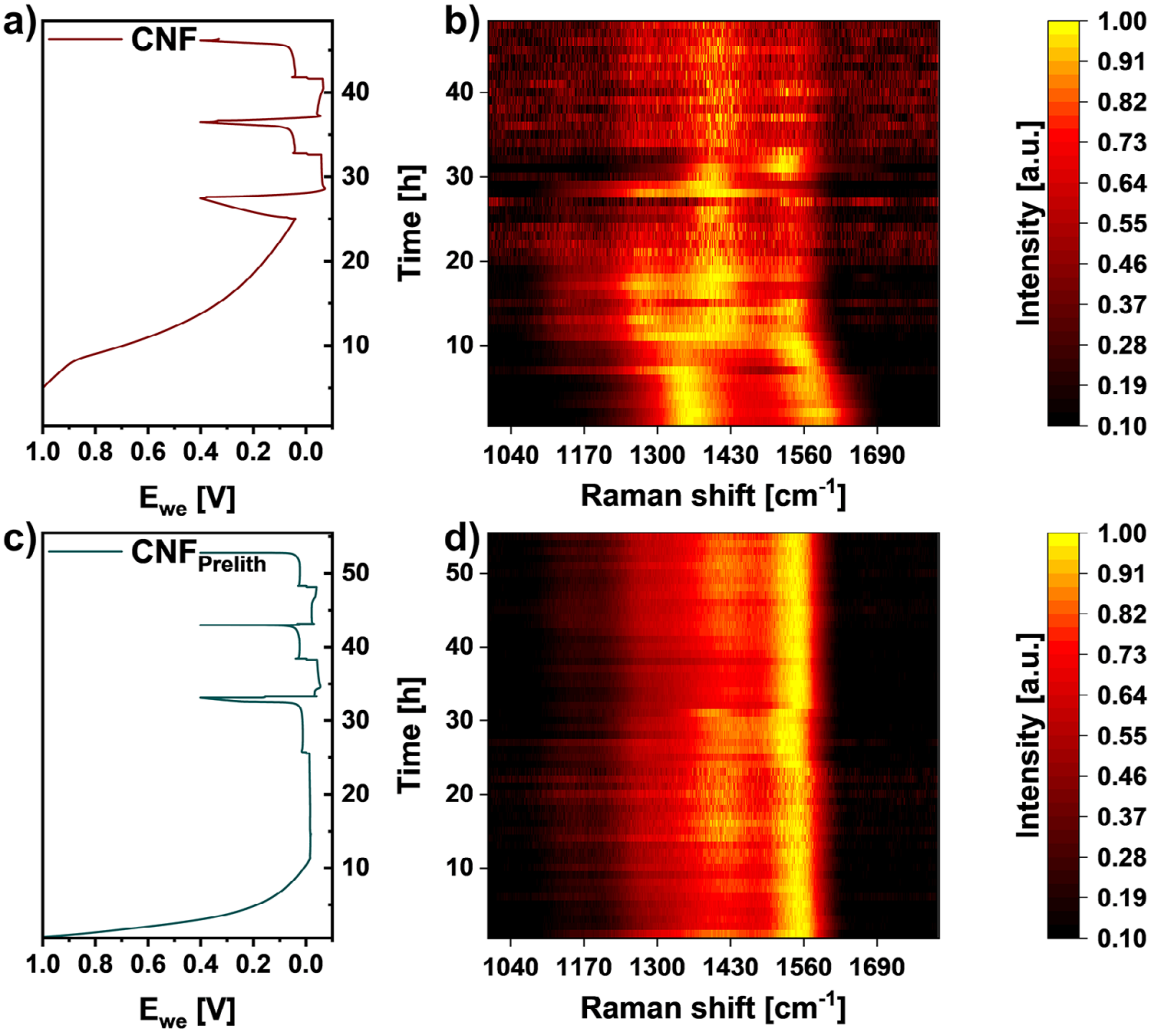
(a) and (c) Voltage vs time profile for CNF and CNF_Prelith_ in an in situ Raman coin cell equipped with a glass window, conducted at an areal capacity of 2.5 mA cm^−2^. (b) and (d) Intensity heatmaps from in situ Raman spectroscopy of CNF and CNF_Prelith_, respectively.

Further insights into the superior cycling stability of CNF_Prelith_ were obtained from in situ Raman spectroscopy, with the electrochemical data from the in situ cell shown in Figure 4a, and the Raman data for CNF in Figure 4b and for CNF_Prelith_ in Figure 4c. While the absolute electrochemical metrics in the in situ cell differ from those in coin cells due to setup constraints (e.g., stack pressure), the qualitative trends and stability evolution remain consistent, validating the mechanism. This also indicates that the system may benefit from pressure optimization. For the CNF interlayer, a pronounced red shift of the G‐band is observed within the first few hours, suggesting progressive lithiation of the carbon framework. This redshift is caused by the combined effects of lithium‐induced n‐doping and the tensile strain of the graphene during lithiation [36, 37]. The increased intensity of the D3‐band around 1460 cm^−1^ after approximately 10 h indicates enhanced surface‐related disorder, likely caused by decomposition products, which correlates with the continuous electrolyte consumption observed in the voltage profile. This is consistent with the electrochemical behavior, where the cell potential reached approximately 0.9 V after 10 h, corresponding to the potential at which the electrolyte begins to decompose to form a SEI. Even beyond this stage, the CNF‐based cell did not reach a steady state, as continuous spectral evolution was observed over three consecutive cycles, reflecting ongoing surface and structural degradation. In contrast, the intensity heatmap for CNF_Prelith_ displays minimal spectral variations. Unlike for the pristine CNF, the G‐band position remains locked at approximately 1550 cm^−2^ without observable redshifts, and the D/G intensity ratio shows negligible fluctuation throughout the lithiation/delithiation process. The absence of a red shift during first lithiation in the G‐band can be explained during the prelithiation, where defect sites of the CNF were already preoccupied by Li‐ions demonstrated by the existing shift of 40 cm^−1^ between the uncycled state of CNF and CNF_Prelith_. As illustrated in Figure S11, the in situ Raman spectra of CNF_Prelith_ exhibits enhanced stability across multiple cycles, with only slight changes between the pristine, lithiated, and delithiated states over the three measured cycles. The absence of significant intensity changes or band shifts highlights the improved mechanical stability provided by prelithiation and the passivating effect of the artificial SEI.

Consequently, the prelithiated interlayer maintains its structural and electronic integrity during repeated cycling, rationalizing the significantly enhanced electrochemical performance observed in the preceding sections.

**FIGURE 5:**
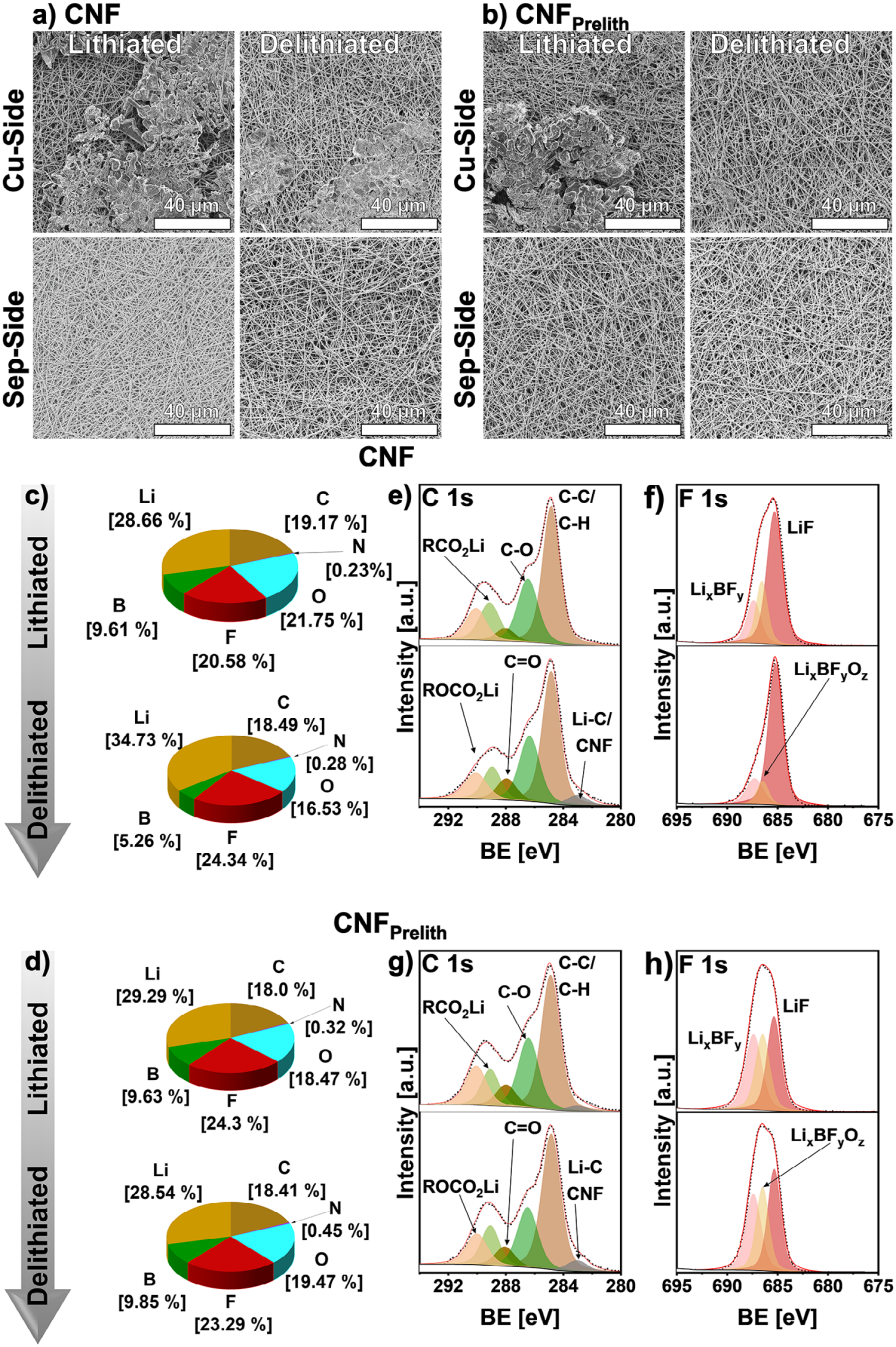
SEM images of CNF (a) and CNF_Prelith_ (b) depicting the lithiated (left) and delithiated states (right) for the side facing the separator (left) and the side facing the Cu foil (right). Distribution of the elements C, N, O, F, B, and Li for CNF and CNF_Prelith_ (c) and (d), *C 1s* and *F 1s* XPS spectra for CNF and CNF_Prelith_. (e)–(g) The spectra are referenced to 284.8 eV for the C─C/C─H signal. All spectra are normalized, with the highest signal in each spectrum set to 1. Survey measurements are provided in Figure S12. Detailed measurement parameters are listed in Table S2. The fitting parameters are listed in Table S5.

To gain insights into the morphological stability of the lithium‐metal interface during cycling, SEM analyses were performed after five full plating and stripping cycles. The efficacy of the 3D carbon‐based interlayers in suppressing top‐growth phenomena was evaluated in particular. This top‐growth behavior is commonly associated with dendritic lithium formation and poor reversibility in lithium‐metal batteries. SEM investigations were carried out on both the Cu‐facing and separator‐facing sides of the interlayers in both lithiated and delithiated states, as illustrated in Figure 5a,b.

SEM images of the pristine CNF interlayer after cycling reveal a distinct asymmetry in lithium deposition. In the lithiated state, metallic lithium is predominantly observed on the side facing the copper current collector, while the separator‐facing surface remains largely free of visible lithium deposits. The internal architecture of the 3D network serves as a physical constraint, discouraging lithium penetration toward the separator and mitigating the risk of top‐growth and dendritic propagation.

Despite this seemingly favorable lithium distribution, residual metallic lithium persists on the Cu‐facing side of the CNF interlayer in the delithiated state. These remnants indicate that a portion of the plated lithium becomes electronically disconnected from the current collector during stripping, forming electrically isolated “dead lithium” within the fiber matrix. Such irreversible lithium accumulation is detrimental to long‐term cycling, contributing to continuous lithium loss, decreased Coulombic efficiency, and increased risk of uncontrolled dendritic growth during subsequent cycles. The presence of these isolated lithium clusters may accelerate the onset of morphological instabilities, exacerbating interfacial degradation over extended cycling.

In contrast, the SEM images of the prelithiated interlayer CNF_Prelith_ reveal a significantly different behavior. In the lithiated state, lithium is again localized at the Cu‐facing interface, with the separator‐facing side remaining free of deposits, confirming that CNF_Prelith_ retains the ability to suppress top‐growth behavior during electrochemical cycling. However, in the delithiated state, a critical distinction is observed: no residual metallic lithium is detected on the CNF_Prelith_ surface. This indicates nearly complete lithium removal during stripping, reflecting superior reversibility compared to pristine CNF. The observed reversibility is consistent with the high first‐cycle Coulombic efficiency of 96.3 % and the prolonged cycling stability demonstrated for CNF_Prelith_ based cells.

The enhanced lithium reversibility observed via SEM aligns well with in situ Raman and XPS findings, which indicate good structural stability of both the carbon framework and the SEI in CNF_Prelith_. These results demonstrate that the chemically engineered interlayer provides a stable electrochemical environment for lithium plating and stripping, promoting homogeneous lithium nucleation while minimizing the formation of electronically isolated lithium domains. Furthermore, the improved mechanical integrity of the prelithiated interlayer likely contributes to stronger contact retention between plated lithium and the current collector during cycling, further suppressing the generation of inactive lithium and enhancing long‐term interfacial stability.

To further investigate the chemical stability and compositional evolution of the SEI formed on CNF‐based interlayers, XPS analyses were conducted after an initial formation cycle and five additional full plating/stripping cycles. Measurements were performed in both lithiated and delithiated states, capturing the surface composition after lithium deposition and removal. Elemental and chemical state information extracted from these spectra is presented in Figure 5c–h.

Figure 5c and d show the atomic concentrations of key elements on the surfaces of CNF and CNF_Prelith_ electrodes in both states. For CNF, comparison of lithiated and delithiated states reveals pronounced variations in oxygen, fluorine, and lithium, while carbon content remains relatively stable (19.17 at.% vs. 18.49 at.%). These changes indicate continuous SEI evolution and ongoing electrolyte decomposition, consistent with in situ Raman observations showing dynamic surface transformations as well as the formation of dead lithium observed in the SEM‐images. In contrast, CNF_Prelith_ exhibits compositional stability, with negligible variations between lithiated and delithiated states. Together with the clean, compact surface observed in the SEM images of the delithiated states, this confirms that the SEI effectively contracts and the lithium is fully extracted.

The presence of fluorine‐ and boron‐rich species on CNF_Prelith_ likely originates from initial reactions between the prelithiated carbon and the electrolyte, where the prelithiated surface promotes reductions of anions in the electrolyte (BF_4_^−^ and DFOB^−^), contributing to an inorganic‐rich SEI with improved ionic conductivity and mechanical robustness [50, 51]. These results indicate that the prelithiated surface acts as a chemical precursor, facilitating the formation of a more stable, inorganic‐dominated SEI rather than serving as the final passivation layer.

High‐resolution *C 1s* spectra (Figure 5c,d) provide further insight into surface‐bound species. In both CNF and CNF_Prelith_ samples, six distinct carbon environments are identified. The dominant peak at 284.8 eV corresponds to C─C/C─H bonds, characteristic of organic SEI components. Peaks at 286.5 eV and 288.0 eV correspond to C─O and C═O functionalities, respectively. Peaks at 289.0 eV and 290.3 eV are assigned to RCO_2_Li and ROCO_2_Li species, decomposition products of cyclic carbonate electrolytes. In addition, a minor peak near 283 eV can be observed. This peak can be attributed to C─Li bonds and highlights the interaction between Li and the carbon. However, it could also be attributed to CNF from the bulk material, since a potential gradient has been formed between the SEI and the bulk. This causes components of the SEI to shift to higher binding energies. Due to referencing a component of the SEI with C─C/C─H it can appear that the bulk material has been shifted to lower binding energies. In CNF, slight increases in ROCO_2_Li and RCO_2_Li during cycling suggest ongoing electrolyte decomposition and surface instability, whereas CNF_Prelith_ displays minimal variation, reflecting a more stable SEI formation.

High‐resolution *F 1s* spectra (Figure 5e,f) reveal three fluorine‐containing species of LiF at 685.3 eV, Li_x_BF_y_O_z_ at 686.4 eV, and Li_x_BF_y_ at 687.3 eV [52, 53]. These species result from the decomposition of lithium salts (LiBF_4_ and LiDFOB) and the FEC additive. For CNF, significant differences in LiF concentration are observed between lithiated (13.14 at.%) and delithiated (18.03 at.%) states, indicating ongoing SEI evolution. In contrast, CNF_Prelith_ exhibits negligible changes, suggesting that the fluorinated species formed during the initial cycles remain stable, providing a mechanically robust and ionically conductive SEI.

Additional insights from high‐resolution *N 1s*, *O 1s*, *B 1s*, and *Li 1s* spectra (Figure S13) confirm that CNF undergoes noticeable compositional changes during cycling, including the formation of borate‐ and oxide‐based species. CNF_Prelith_, however, exhibits only minor variations, indicating a chemically stable SEI framework. Stable *B 1s* signals further support the formation of a durable inorganic backbone. Collectively, these findings suggest that prelithiation not only facilitates early‐stage lithium nucleation but also directs electrolyte decomposition pathways to favor robust, inorganic SEI formation. The artificial SEI initially formed on CNF_Prelith_ serves as a nucleation and chemical template, guiding the evolution of a high‐integrity SEI rather than acting as the final passivation layer itself.

**FIGURE 6:**
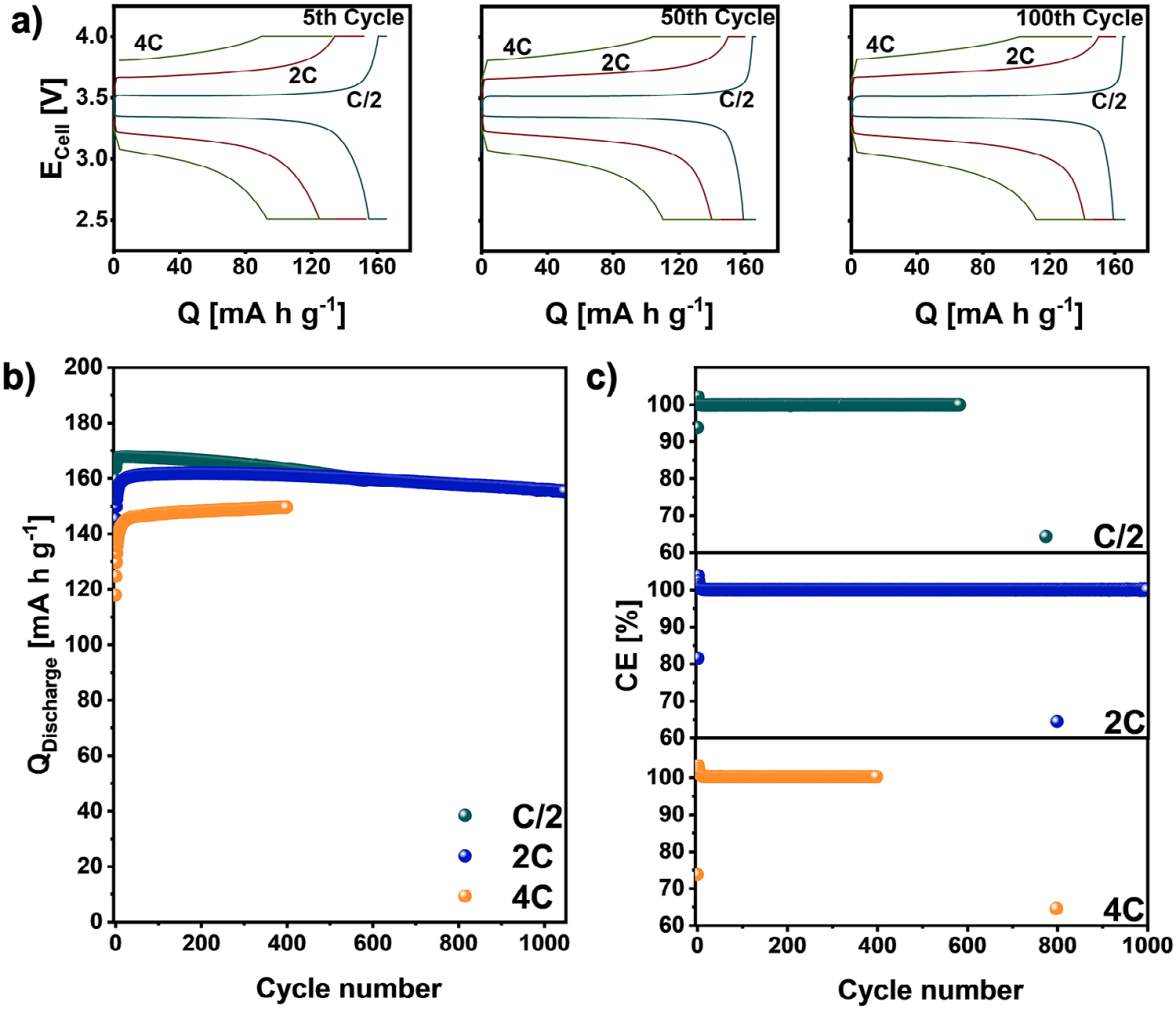
(a) Charge–discharge curves vs. capacity profiles from the 5th, 50th, and 100th cycle of CNF_Prelith_ vs. LFP at 0.5, 2, and 4 C, respectively. (b) Long‐term cycling discharge capacities and (c) Corresponding CE for the cells shown in (b).

To further evaluate the electrochemical stability and practical applicability of the prelithiated carbon fiber interlayer (CNF_Prelith_), ZELMBs employing LFP as the cathode were assembled and subjected to long‐term cycling under varying current densities, as illustrated in Figure 6. Here, “*zero‐excess* lithium metal” refers to an anode‐side configuration without Li metal foil, while a limited amount of lithium is introduced through the prelithiated CNF interlayer. Importantly, no extended formation protocol was applied in these experiments, providing a stringent assessment of the interlayer's performance under nonideal conditions. The absence of a preconditioning protocol emphasizes the intrinsic ability of CNF_Prelith_ to stabilize the lithium‐metal interface and maintain consistent cell performance from the very first cycle. Pristine CNF was not evaluated under these conditions because the LFP cathode used in the full‐cell tests provides a capacity of 1.25 mAh cm^−2^, whereas plating/stripping experiments indicate that more than 2.375 mAh cm^−2^ is required to achieve reversible lithium plating on pristine CNF. Therefore, under the applied conditions, pristine CNF is not expected to enable stable operation. While the bare Cu current collector exhibits a high initial Coulombic efficiency in lithium‐metal plating/stripping tests, its cycling stability remains limited, leading to rapid performance decay as depicted in Figure 3. A similar trend is observed under *zero‐excess* lithium metal conditions, where, despite an initial conditioning step at 0.05 C (two cycles) followed by cycling at 0.1 C, unstable lithium deposition persists during the early cycles. In particular, dendritic growth is observed within the first few cycles, even though a high Coulombic efficiency above 95% is maintained, as shown in Figure S14. Therefore, the bare Cu current collector was not investigated at higher C‐rates under the present conditions.

Figure 6a presents representative voltage–capacity profiles for cells cycled at 0.5, 2, and 4 C, displayed at the 5th, 50th, and 100th cycles. Even at the 5th cycle, clear differences in polarization behavior emerge as a function of the applied C‐rate.

The cell cycled at 0.5 C exhibits a relatively flat discharge plateau around 3.34 V, indicative of low internal resistance and efficient lithium‐ion transport across the electrode‐electrolyte interface. In contrast, the cells cycled at higher C‐rates display a pronounced increase in overpotential. At 2 C, the discharge voltage drops to approximately 3.18 V, and at 4 C it further decreases to 3.01 V, reflecting the kinetic limitations associated with fast lithium plating and stripping.

Interestingly, the evolution of overpotential over extended cycling reveals complex, adaptive behavior. While the low‐rate 0.5 C cell maintains a stable voltage profile over 580 cycles with only negligible changes, the 2 C cell exhibits a gradual reduction in overpotential between the 5th and 100th cycles. Notably, the 4 C cell demonstrates a gradual interfacial stabilization process. As shown in Figure 6a, the voltage hysteresis, defined as the potential difference between the charge and discharge plateaus, visibly narrows from the 5th to the 100th cycle. The discharge plateau, initially suppressed at ∼3.01 V and gradually shifts upward and flattens in subsequent cycles. This trend in the cells cycled at 2 C and 4 C demonstrates limitations of the system, as activation and wetting problems have to be overcome without an extended formation protocol.

Long‐term capacity retention under these conditions is shown in Figure 6b, while the corresponding CEs are presented in Figure 6c. The 0.5 C cell achieves a discharge capacity of 167.7 mAh g^−1^ after 25 cycles, which decreases slightly to 159.4 mAh g^−1^after 580 cycles, corresponding to a capacity retention of 95.0%. The Coulombic efficiency remains consistently above 99.97% throughout, indicating minimal side reactions or lithium loss and confirming the stability of the CNF_Prelith_ interlayer under moderate cycling conditions. However, at the second cycle, the CE reached 102.07% and stepwise decreased to 100.02% for the 6th cycle. This behavior may be attributed to several factors. One possible explanation is the initial cracking of carbon components in the LFP cathode, which could enable increased utilization of active material. In addition, a redistribution of lithium stored in the prelithiated CNF interlayer may contribute to the apparent excess capacity [54, 55].

The cell cycled at 2 C achieves a capacity of 160.5 mAh g^−1^ after 25 cycles, equivalent to 95.73% of the 0.5 C capacity. After 1050 cycles, the capacity remains at 155.2 mAh g^−1^, corresponding to 97.5% retention. The high‐capacity retention and high average CE of 99.93%, despite increased kinetic constraints, underscore the ability of CNF_Prelith_ to maintain a stable interface under fast‐charging conditions. In the first cycles, the same behavior as for the 0.5 C cell can be observed as the CE in the second cycle reaches 103.80% and stepwise decreases to 100.02% in the 12th cycle after which it decreases below 100%. The interlayer efficiently accommodates higher rates of lithium plating and stripping without promoting inactive lithium accumulation or SEI degradation.

A different trend is observed in the cell directly cycled at 4 C. Despite the harsh electrochemical environment, the initial capacity reaches 144.8 mAh g^−1^ after 25 cycles, corresponding to 86.36% of the 0.5 C capacity. Notably, the capacity progressively increases over subsequent cycles, reaching 149.4 mAh g^−1^ after 400 cycles, corresponding to 103.2% of the initial capacity. This unusual capacity increase may arise from multiple factors. Possible contributions include cathode activation and wetting processes, as well as normalization, balancing, or utilization effects under high‐rate conditions without an extended formation protocol. In addition, similar behavior observed in the 0.5 C and 2 C cells, where Coulombic efficiencies exceeding 100% (e.g., 103.02% in the second cycle) were recorded, suggesting that these effects may not be limited to a single mechanism. Instead of falling below 100% like in the other cells, it remains above 100% for the entire 400 cycles, explaining the increase in capacity retention. However, after approximately 200 cycles, the overpotential starts to rise again. Due to this rise in overpotential, cycling beyond 400 cycles was not feasible, highlighting the limitations imposed by extremely fast‐charging conditions without an extended formation protocol.

## 3 Conclusions

In summary, this work demonstrates a practical strategy to overcome the critical stability bottlenecks of *zero‐excess* lithium metal batteries by introducing a chemically prelithiated carbon nanofiber (CNF_Prelith_), wherein prelithiation introduces a small amount of lithium into the host structure. Unlike conventional interlayers, which suffer from initial active lithium consumption and unstable interface evolution, our prelithiation protocol creates a multifunctional host that addresses these issues simultaneously.

Multimodal characterization reveals that the reaction with *n*‐butyllithium not only dopes the carbon lattice, as evidenced by the redshifted G‐band and increased structural disorder, but also forms a robust, inorganic‐rich artificial solid electrolyte interphase composed of LiH, LiOH, Li_2_O, as well as stable organic salts. This engineered interface proves critical in lowering the lithium nucleation overpotential, homogenizing Li^+^ flux, and effectively suppressing the top‐growth of dendrites, as confirmed by postcycling SEM and in situ Raman spectroscopy.

Consequently, full cells paired with LiFePO_4_ cathodes exhibited high electrochemical performance. The CNF_Prelith_ interlayer enabled high‐capacity retention of 95.0% at 0.5 C and 97.5% at 2 C (over 1000 cycles) with high CEs. Most notably, the system displayed pronounced robustness under aggressive 4 C fast‐charging conditions. Crucially, these performance metrics for the ZELMBs configuration were achieved without an extended formation protocol, a step that is typically mandatory but time‐consuming in standard manufacturing.

Ultimately, this study demonstrates that surface chemical engineering via prelithiation can improve the stability of the lithium metal interface against kinetic and chemical degradation. In addition, it may offer insights into strategies that simplify interfacial design in lithium‐metal battery systems. This work provides perspectives on potential pathways toward translating high‐energy‐density concepts into lithium‐metal battery architectures under laboratory‐scale conditions

## 4 Experimental Section

### 4.1 Synthesis of CNF

Polyacrylonitrile (PAN, 3.776 g) was dissolved in 40 mL N,N‐dimethylformamide (DMF) and stirred at room temperature for 24 h to obtain a homogeneous solution. The precursor solution was electrospun using an IME Medical Electrospinning system (The Netherlands) under controlled conditions (25°C, 30% relative humidity). Electrospinning was carried out at a flow rate of 20 µL min^−1^, with the needle moving parallel to the collector at 20 mm s^−1^ and a turn delay of 500 ns. The collector rotated at 700 rpm, and the process continued for ∼6.5 h until 8 mL of solution had been consumed. The resulting nanofiber mat was stabilized at 250°C for 15 h in air, using a heating rate of 5°C min^−1^. Subsequently, the fibers were reduced at 500°C for 2.5 h under an Ar/H_2_ (97:3) atmosphere, followed by carbonization at 700°C under Ar. Circular discs with an area of 0.95 cm^2^ were then punched from the carbon nanofiber (CNF) mats for electrochemical testing.

### 4.2 Synthesis of CNF_Prelith_

Prelithiation of CNF discs was performed entirely under inert conditions. The discs were immersed in 1 mL of *n*‐BuLi solution and kept at room temperature for 1 h. After removal of excess reagent, the discs were heated at 500°C for 20 min, yielding prelithiated CNF (CNF_Prelith_).

### 4.3 Structural Characterizations

The phase composition of the synthesized electrode materials was studied by x‐ray Diffraction (XRD) on a STOE STADI‐P setup with Mo‐K*α*1 radiation (λ = 0.70932 Å) and transmission geometry. Prior to testing, the samples were handled inside the glovebox, where they were placed in a special air‐tight holder with Kapton windows to prevent any interaction with the ambient air. The measurements were performed in the 5–45 range of 2θ, using a linear MYTHEN2 detector and Ge(111) monochromator.

Scanning transmission electron microscopy (STEM) was conducted on an FEI Titan G2 80–200 (FEI/Thermo Fisher Scientific, USA) equipped with a Cs‐probe corrector and a HAADF detector. The microscope was operated at 200 kV with a probe semi‐angle of 24.7 mrad. Elemental distribution maps were obtained by energy‐dispersive X‐ray spectroscopy (EDS) using four large‐solid‐angle silicon drift detectors.

BET measurements were performed to determine the surface area via Ar adsorption, using the automated adsorption analyzer Micro 30°C‐02‐Analysis Station (3P Instruments, Germany). Monte Carlo (MC) simulations were carried out to quantify the pore volume obtained from Ar adsorption, employing the Quantachrome ASiQwin Automated Gas Sorption Data software (Quantachrome, Germany). In situ Raman measurements were performed using a LabRAM HR Evolution spectrometer (HORIBA Scientific) equipped with a solid‐state laser operating at 532 nm, with 25% laser power. The sample configuration and custom‐designed coin cells were specifically adapted for in situ measurements. The optical access to the coin cells was provided via a 160 µm‐thick glass window that sealed the transmitting hole. The interlayer was positioned on a copper current‐collector ring with a central aperture [56]. Cell cycling was conducted following the electrochemical protocol described in the corresponding section, at a current density of 2.5 mA cm^−2^. The spectral parameters were determined by curve fitting in Origin (OriginLab Corporation, United States) and were deconvolved into five bands using the method proposed by Brubaker et al. [57]. A Pseudo‐Voigt function was used for the D1, D2, and G bands, while the D3 and D4 bands were fitted considering only Gaussian contributions.

Scanning electron microscopy (SEM) was performed using an FEI Quanta FEG 650 (FEI/Thermo Fisher Scientific, USA) equipped with an EDAX‐Octane 70 mm^2^ EDS detector (EDAX‐Ametek, USA). A K&W transfer module (Kammrath & Weiss, Germany) enabled air‐free transfer of samples directly from the glovebox to the SEM chamber. Images were acquired at an acceleration voltage of 2 kV and a spot size of 1, ensuring minimal electron‐beam‐induced damage. To determine the average fiber diameter, 200 individual fibers were measured using the software *ImageJ*. The obtained values were subsequently analyzed using *OriginPro 2024*, where a histogram of the fiber diameter distribution was generated. The average fiber diameter was determined by fitting the data with a lognormal distribution function [58].

The NMR measurements were conducted on a Bruker 800 MHz Avance Neo spectrometer equipped with a 3.2 MAS probe at 20°C. The MAS spinning rate was set to 20 kHz for all measurements. The ^7^Li MAS NMR spectra were referenced to LiF at −1.0 ppm, and a recycle delay of 1500 s was employed [59]. For the CPMAS measurement, a contact time of 200 µs was used along with a proton power ramp covering 70%–100% of the maximum power. A total of 8 dummy scans and 16 scans were acquired for the CPMAS and the thermal measurement. During the inversion recovery experiment, one scan was acquired for each of the 22 delays listed in Table S6. The Inverse Laplace Transform was performed using the IltPy version 1.0.0 [60]. Python 3.13.9 was used to handle all NMR data that were exported and subsequently visualized using Origin. The data was prepared for ILT by choosing 200 data points in the chemical shift dimension from 22.2 ppm to −26.6 ppm, spaced by roughly 0.24 ppm. The first 40 points were used to obtain the standard deviation of the noise to scale the data. The output sampling vector in the *T*_1_ dimension was defined from 10^−3^ to 10^6^ s with 110 logarithmically spaced data points. The initialization was performed using an exponential kernel. Using the previously obtained residuals, the process was repeated for the weighted inversion. For the *T*_1_ heat map, the ILT intensity *g* was scaled by plotting ‐sign(*g*)∙|*g*|^0.5^ to emphasize smaller contributions. The random residuals (Figure S15) demonstrate the validity of the ILT results.

X‐ray photoelectron spectroscopy (XPS) was performed on a Kα spectrometer integrated with a glovebox (Thermo Fisher Scientific, USA). Measurements were carried out under a base pressure of 10^−9^ mbar using an Al‐Kα X‐ray source. Survey spectra were collected at 200 eV pass energy with a spot size of 400 µm. High‐resolution scans were acquired at the same spot and spot size, with a 50 eV pass energy and a 0.1 eV step size. Depth profiling was performed after surface measurements by sputtering a 2 × 1 mm spot with 500 eV Ar^+^ ions. Additional measurements were collected after sputter times of 10, 500, 1041, and 2002 s.

The XPS data were processed using Avantage software (Thermo Fisher Scientific, USA). Quantification was based on instrument‐specific sensitivity factors provided in Avantage, with an additional energy compensation factor applied to account for inelastic scattering. A “smart background,” derived from a Shirley‐type function with the restriction that it remains lower than the measured signal, was used for core‐level peak fitting. Voigt profiles (70% Gaussian, 30% Lorentzian) were applied. The spectra for CNF are referenced to 398.0 eV for the pyridinic nitrogen, and the spectra of CNF_Prelith_ as well as the cycled samples, are referenced to 284.8 eV for the signal corresponding to C─C/C─H. For cycled samples, XPS measurements were performed after five charge–discharge cycles with a current density of 0.5 mA cm^−2^, following the electrochemical protocol described in the corresponding section for 2.5 mAh cm^−2^. Cycled samples were washed three times with DEC prior to drying and XPS measurements.

### 4.4 Electrochemical Measurements

Plating/stripping and impedance tests were performed in CR2032 coin cells with a Cu foil/sample‖electrolyte‖Li configuration. A Celgard 2400 separator was used together with an electrolyte consisting of 0.6 M LiBF_4_ and 0.6 M LiDFOB dissolved in FEC/DEC (1:2 by volume). The tests were initiated with a formation plating step at 0.05 or 0.1 mA cm^−2^ for 25 h, followed by stripping at the same current density to 0.4 V. Subsequently, cycling was carried out at 0.25 or 0.5 mA cm^−2,^ maintaining an areal capacity of 2.5 mA cm^−2^

For the critical current tests, the same cell configuration was used. After two formation cycles at 0.05 or 0.1 mA cm^−2^ the current was stepwise increased every 5 cycles till cell failure. The areal capacity was either fixed at 1.25 or 2.5 mA cm^−2^

*Zero‐excess* Li metal batteries (Cu foil/sample‖electrolyte‖LFP) were assembled in CR2032 coin cells and cycled at different current densities within a cutoff voltage window of 2.5–3.8 V vs. Li^+^/Li, followed by a constant‐voltage step until the current decayed to 10% of its initial value. LFP electrodes (NEI Corporation, USA) with an active mass loading of 5 ± 0.4 mg cm^−2^ were punched into 10 mm disks and employed as cathodes. The same separator and electrolyte as in the plating/stripping tests were used.

All electrochemical measurements were conducted at 25°C using multichannel potentiostats (VMP3/MPG‐2, BioLogic, France) housed in a temperature‐controlled chamber (Binder, Germany).

## Conflicts of Interest

The authors declare no conflicts of interest.

## Supporting information




**Supporting File**: advs75690‐sup‐0001‐SuppMat.docx.

## Data Availability

The data that support the findings of this study are available from the corresponding author upon reasonable request.
