# Supplementary material for: Enhanced Stability in Zero‐Excess Li‐Metal Batteries via Prelithiated Carbon Nanofiber Interlayers
Source: Adv Sci (Weinh). 2026 May 14:e75690. Online ahead of print. doi: 10.1002/advs.75690 (PMC13335860; doi:10.1002/advs.75690)
Supplement: Supplementary file 1 — Supporting File: advs75690‐sup‐0001‐SuppMat.docx. [file ADVS-9999-e75690-s001.docx]

**Enhanced Stability in Zero-Excess Li-Metal Batteries via Prelithiated Carbon Nanofiber Interlayers**

Sandro Schöner^1,2^, Marius Ast^3^, Vera Michaela Barysch^1,4^, Rebecca Erkes^1,2^, Jule Meier-Merziger^1,2^, Pengfei Cao^5^, Joachim Mayer^5,6^, Josef Granwehr^1,4^, Fabian Jeschull^3^, Hermann Tempel^1^, Shicheng Yu^1^* and Rüdiger-A. Eichel^1,2,7^

^1^S. Schöner, V. M. Barysch, R. Erkes, J. Meier-Merziger, J. Granwehr, H. Tempel, S. Yu, R.-A. Eichel

Institute of Energy Technologies - Fundamental Electrochemistry (IET-1),

Forschungszentrum Jülich, 52428 Jülich, Germany

E-mail: [s.yu@fz-juelich.de](mailto:s.yu@fz-juelich.de)

^2^ S. Schöner, R. Erkes, J. Meier-Merziger, R.-A. Eichel

Institute of Physical Chemistry - Material and Processes of Electrochemical Energy Storage and Conversion,

RWTH Aachen University, 52074 Aachen, Germany

^3^M. Ast, F. Jeschull

Karlsruher Institute of Technologie (KIT), Institute for Applied Materials (IAM), 76344 Eggenstein Leopoldshafen, Germany

^4^V. M. Barysch, J. Granwehr

Institute of Technical and Macromolecular Chemistry

RWTH Aachen University, 52074 Aachen, Germany

^5^P. Cao, J. Mayer

Ernst Ruska-Centre for Microscopy and Spectroscopy with Electrons,

Forschungszentrum Jülich, 52428 Jülich, Germany

^6^J. Mayer

Central Facility for Electron Microscopy (GFE),

RWTH Aachen University, 52064 Aachen, Germany

^7^R.-A. Eichel

Faculty of Mechanical Engineering

RWTH Aachen University, 52062 Aachen, Germany

Keywords: prelithiation, artificial SEI, *zero-excess* Li metal battery, Li deposition, 3D interlayer, carbon nanofiber


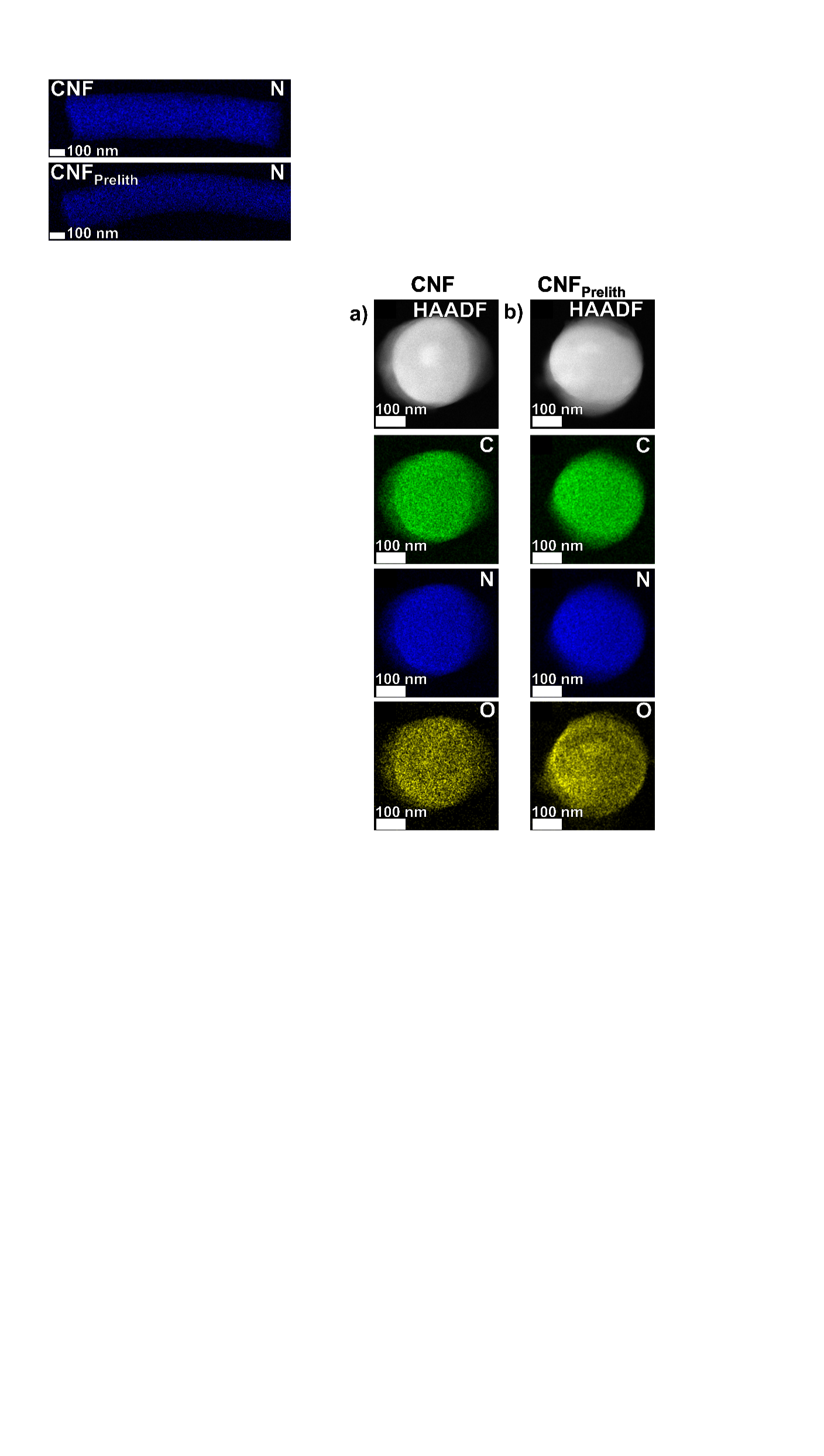


**Figure S1** STEM-EDX elemental mapping of N for CNF and CNF_Prelith_. The corresponding STEM images and STEM-EDX elemental mappings of C and O are depicted in **Figure 1** of the main text.


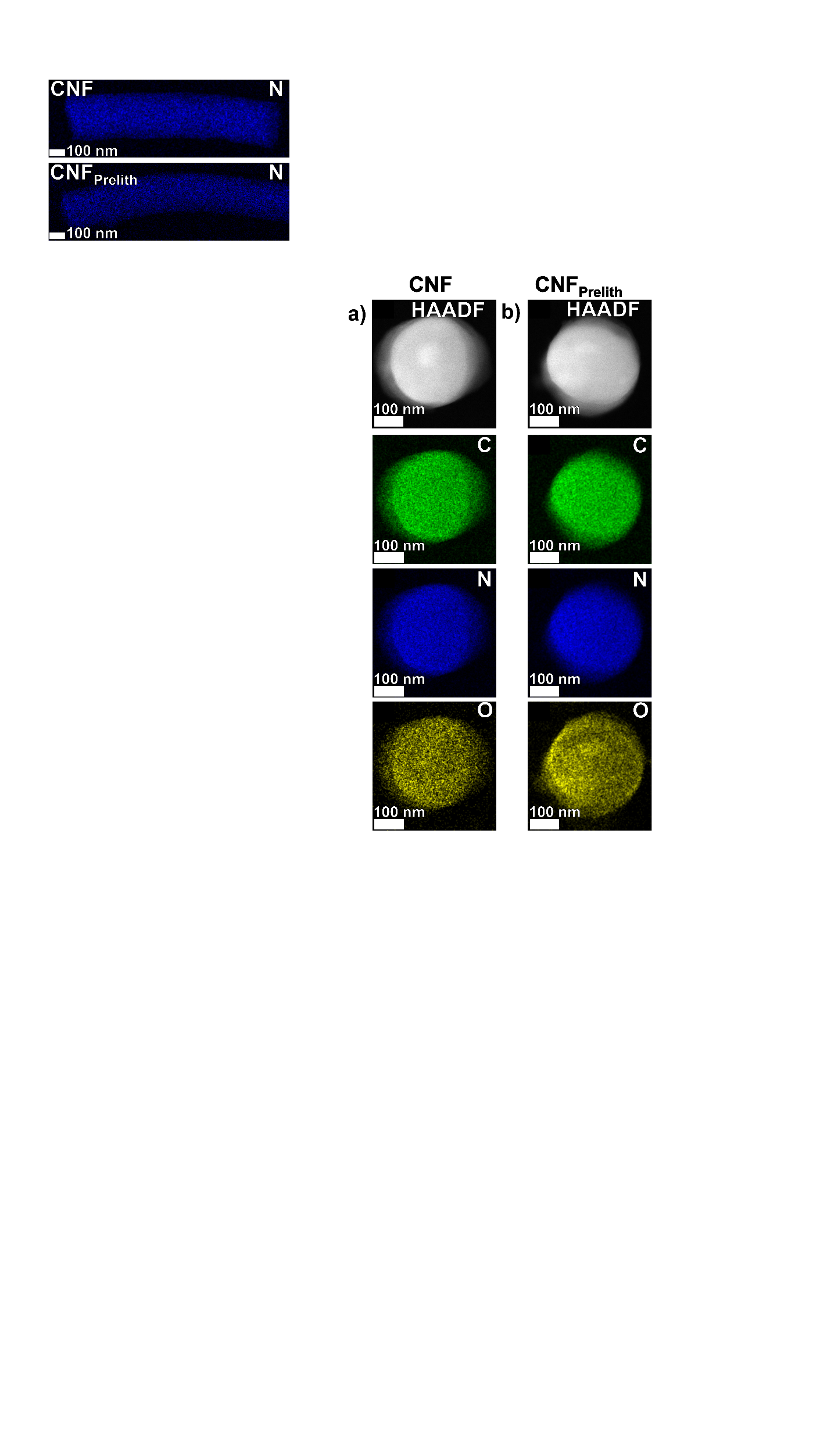


**Figure S2** Cross-section STEM images and STEM-EDX elemental mappings of a) CNF and b) CNF_Prelith_ showing the field of view and the element distribution of C, N, and O.

**Table S1** Spectral parameters for the first-order Raman bands of CNF and CNF_Prelith_, showing the band position (Stokes Raman shift), full width at half maximum (FWHM), the peak area (A), and the peak area ratios.

| Band | Parameter | CNF | CNF_Prelith_ |
| --- | --- | --- | --- |
| G | Position [cm^-1^] | 1,590 | 1,547 |
|  | FWHM [cm^-1^] | 122.59 | 81.87 |
|  | A [cm^2^] | 95.91 | 57.27 |
| D1 | Position [cm^-1^] | 1,359 | 1,300 |
|  | FWHM [cm^-1^] | 138.96 | 170 |
|  | A [cm^2^] | 123.8 | 92.35 |
|  | A_D1_/A_G_ | 1.29 | 1.61 |
| D2 | Position [cm^-1^] | 1,624 | 1,585 |
|  | FWHM [cm^-1^] | 75 | 50 |
|  | A [cm^2^] | 10.14 | 15.31 |
| D3 | Position [cm^-1^] | 1,482 | 1,439 |
|  | FWHM [cm^-1^] | 134.48 | 163.14 |
|  | A [cm^2^] | 70.78 | 128.5 |
|  | A_D3+D4_/A_total_ | 0.33 | 0.46 |
| D4 | Position [cm^-1^] | 1,247 | 1,149 |
|  | FWHM [cm^-1^] | 170 | 96.14 |
|  | A [cm^2^] | 43.56 | 14.70 |
|  | A_D4+D3_/A_total_ | 0.33 | 0.46 |


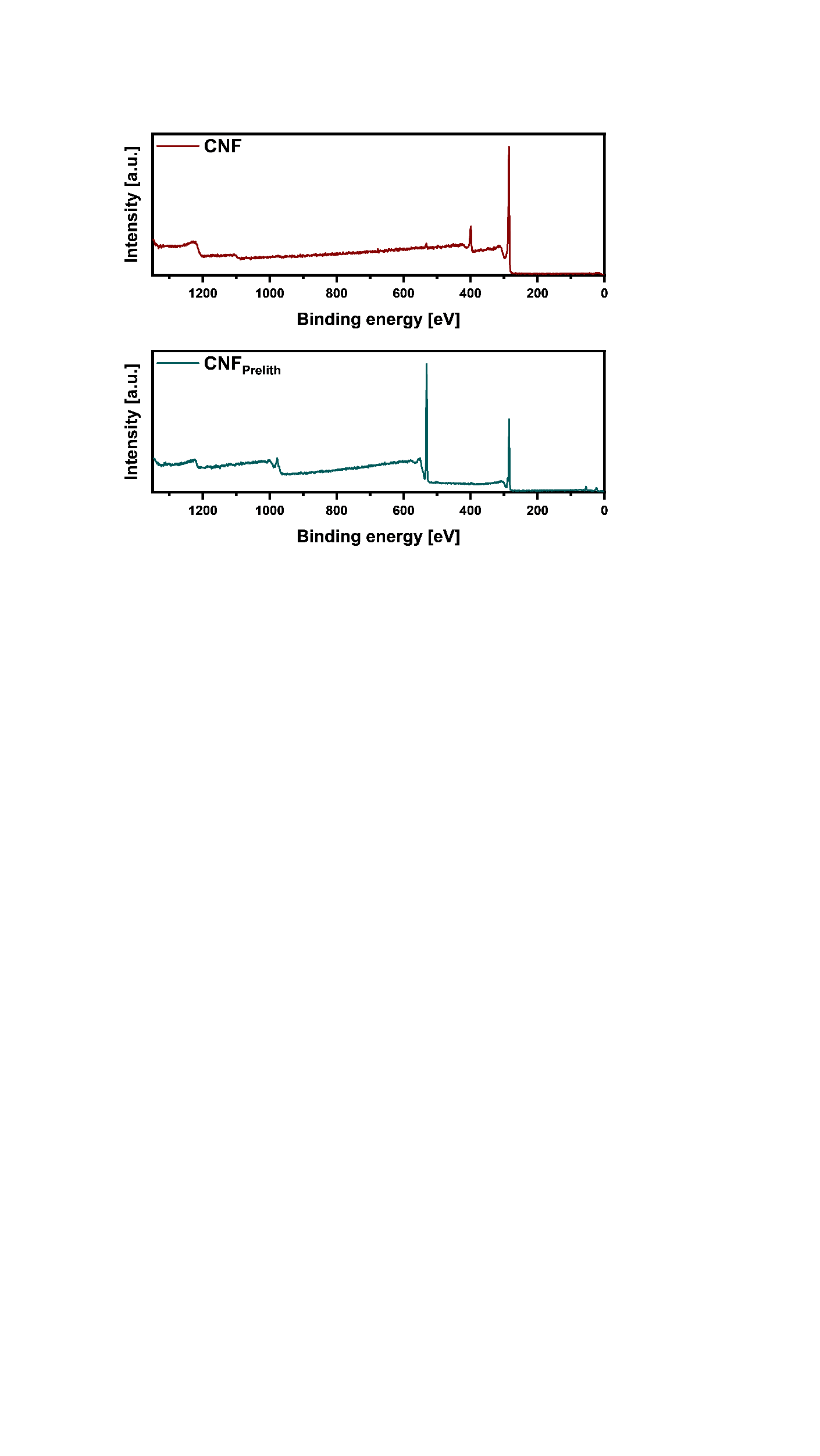


**Figure S3** Survey measurement of the XPS spectrum for CNF and CNF_Prelith_. The spectrum for CNF is referenced to pyridinic N at 398.0 eV.  The spectrum for CNF_Prelith_ is referenced to C-C/C-H at 284.8 eV. All spectra are normalized, with the highest signal in each spectrum set to 1.

**Table S2** Measurement details of the XPS scans used for the uncycled CNF and CNF_Prelith_ as well as for the delithiated and lithiated cycling states of both materials.

| **ID** | **Scans** | **Dwell time [ms]** | **Pass energy [eV]** | **Range**  **[eV]** |
| --- | --- | --- | --- | --- |
| Survey | 10 | 10 | 200 | 10-1350 |
| *C 1s* | 10 | 50 | 50 | 279-310 |
| *O 1s* | 5 | 50 | 50 | 520-545 |
| *Li 1s* | 10 | 50 | 50 | 50-70 |
| *N 1s* | 10 | 50 | 50 | 392-410 |
| *B 1s* | 10 | 50 | 50 | 210-178 |
| *F 1s* | 10 | 50 | 50 | 678-705 |

**Table S3** Quantification results and fitting parameters of different components of CNF on the surface as well as after 10, 500, 1000, and 2000 s of sputter time. Quantification refers to elements highlighted in bold. The BEs of CNF are referenced to pyridinic N at 398.0 eV.

| **Component** | **C** (CNF) | **C**N (**C**-O) | **C**=O | **N** (pyridinic) | -**N**H_2_ | **N** (pyrolic) | **N** (graphitc^1^) | **N** (graphitc^2^ | C-**O** | C=**O** |
| --- | --- | --- | --- | --- | --- | --- | --- | --- | --- | --- |
| **Surface [%]** | 44.81 | 31.91 | 7.1 | 2.79 | 4.71 | 0.97 | 4.11 | 1.94 | 1.02 | 0.64 |
| **Surface BE [eV]** | 284.79 | 285.89 | 287.92 | 398 | 398.73 | 399.7 | 400.44 | 401.5 | 532.98 | 530.97 |
| **Surface FWHM [eV]** | 1.36 | 1.75 | 2 | 1.25 | 1.49 | 1.31 | 1.51 | 1.6 | 2 | 2 |
| **Surface Intensity [CPS]** | 46799.81 | 25908.22 | 5045.99 | 4943 | 6969.03 | 1633 | 6011.26 | 2673.61 | 1758.39 | 1098.39 |
| **10 s sputter time [%]** | 44.88 | 31.96 | 7.12 | 2.8 | 4.72 | 0.97 | 4.11 | 1.94 | 0.86 | 0.64 |
| **10 s sputter time BE [eV]** | 284.79 | 285.89 | 287.92 | 398 | 398.73 | 399.7 | 400.44 | 401.5 | 532.98 | 530.97 |
| **10 s sputter time FWHM [eV]** | 1.36 | 1.75 | 2 | 1.25 | 1.49 | 1.31 | 1.51 | 1.6 | 1.69 | 2 |
| **10 s sputter time Intensity [CPS]** | 46799.81 | 25908.22 | 5045.99 | 4943 | 6969.03 | 1633 | 6011.26 | 2673.61 | 1758.39 | 1098.39 |
| **500 s sputter time [%]** | 44.09 | 35.3 | 8.25 | 2.13 | 3.11 | 1.95 | 3.06 | 1.28 | 0.62 | 0.22 |
| **500 s sputter time BE [eV]** | 284.6 | 285.71 | 287.78 | 398 | 398.67 | 399.69 | 400.43 | 401.5 | 532.98 | 531.02 |
| **500 s sputter time FWHM [eV]** | 1.3 | 1.71 | 2 | 1.31 | 1.4 | 1.53 | 1.6 | 1.6 | 2 | 1.6 |
| **500 s sputter time Intensity [CPS]** | 49088.3 | 29758.4 | 5953.43 | 3655.1 | 4988.47 | 2857.13 | 4295.81 | 1800.68 | 1075.04 | 470.28 |
| **1041 s sputter time [%]** | 46.30 | 33.79 | 7.88 | 1.17 | 4.01 | 1.35 | 2.95 | 1.21 | 0.77 | 0.57 |
| **1041 s sputter time BE [eV]** | 284.58 | 285.68 | 287.72 | 398.00 | 398.63 | 399.70 | 400.40 | 401.50 | 533.15 | 530.80 |
| **1041 s sputter time FWHM [eV]** | 1.30 | 1.63 | 2.00 | 1.29 | 1.57 | 1.57 | 1.53 | 1.60 | 2.00 | 2.00 |
| **1041 s sputter time Intensity [CPS]** | 52351.82 | 30380.71 | 5774.92 | 2073.06 | 5831.99 | 1964.66 | 4387.82 | 1720.92 | 1369.88 | 1017.66 |
| **2002 s sputter time[%]** | 45.47 | 35.70 | 7.55 | 1.40 | 3.52 | 1.12 | 2.93 | 0.93 | 0.73 | 0.64 |
| **2002 s sputter time BE [eV]** | 284.57 | 285.65 | 287.72 | 398.00 | 398.63 | 399.70 | 400.40 | 401.50 | 533.44 | 530.78 |
| **2002 s sputter time FWHM [eV]** | 1.30 | 1.71 | 2.00 | 1.60 | 1.57 | 1.31 | 1.57 | 1.60 | 2.00 | 2.00 |
| **2002 s sputter time Intensity [CPS]** | 53247.13 | 31659.21 | 5736.51 | 2065.46 | 5306.97 | 2025.33 | 4418.95 | 1367.86 | 1347.28 | 1171.60 |

**Table S4** Quantification results and fitting parameters of different components of CNF on the surface as well as after 10, 500, 1000, and 2000 s of sputter time. Quantification refers to elements highlighted in bold. The BEs of CNF_Prelith_ are referenced to C-C/C-H at 284.8 eV.

| **Component** | **C** (C-C/C-H)) | **C**N | R**C**O_2_Li | RO**C**O_2_L**i** | **C**(CNF) | **N^1^** | **N^2^** | C=**O** | Li_2_**O** | **Li** |
| --- | --- | --- | --- | --- | --- | --- | --- | --- | --- | --- |
| **Surface [%]** | 21.15 | 0.77 | 2.70 | 1.50 |  | 0.89 |  | 20.87 | 0.10 | 52.02 |
| **Surface BE [eV]** | 284.80 | 286.16 | 288.75 | 289.90 |  | 398.49 |  | 531.53 | 528.81 | 55.04 |
| **Surface FWHM [eV]** | 1.52 | 1.60 | 1.60 | 1.65 |  | 3.23 |  | 1.60 | 1.02 | 1.81 |
| **Surface Intensity [CPS]** | 23607.59 | 814.78 | 2857.18 | 1530.86 |  | 729.71 |  | 53534.69 | 406.54 | 3402.84 |
| **10 s sputter time [%]** | 17.45 | 0.62 | 2.40 | 1.04 |  | 0.60 |  | 24.58 | 0.39 | 52.91 |
| **10 s sputter time BE [eV]** | 284.80 | 286.16 | 288.75 | 289.90 |  | 398.67 |  | 531.22 | 528.71 | 55.03 |
| **10 s sputter time FWHM [eV]** | 1.55 | 1.60 | 1.57 | 1.31 |  | 2.00 |  | 1.56 | 1.05 | 1.94 |
| **10 s sputter time Intensity [CPS]** | 21419.11 | 738.01 | 2888.76 | 1508.47 |  | 887.55 |  | 72489.35 | 1704.18 | 3625.06 |
| **500 s sputter time [%]** | 13.98 | 2.23 | 1.08 | 1.36 | 8.21 | 1.39 | 1.19 | 12.65 | 6.20 | 51.70 |
| **500 s sputter time BE [eV]** | 284.80 | 286.16 | 288.75 | 290.08 | 283.58 | 399.06 | 397.59 | 531.80 | 528.83 | 55.18 |
| **500 s sputter time FWHM [eV]** | 1.60 | 1.60 | 1.60 | 1.60 | 1.60 | 1.75 | 2.00 | 1.68 | 1.32 | 1.94 |
| **500 s sputter time Intensity [CPS]** | 16364.70 | 2605.71 | 1264.52 | 1588.54 | 9614.79 | 2307.53 | 1736.72 | 33987.96 | 21268.38 | 3490.48 |
| **1041 s sputter time [%]** | 9.10 | 3.23 | 1.57 | 1.49 | 20.12 | 2.25 | 1.92 | 8.52 | 5.76 | 46.05 |
| **1041 s sputter time BE [eV]** | 284.80 | 286.16 | 288.55 | 290.07 | 283.69 | 398.94 | 397.46 | 531.63 | 528.66 | 54.90 |
| **1041 s sputter time FWHM [eV]** | 1.35 | 1.60 | 1.60 | 1.60 | 1.71 | 1.72 | 1.94 | 1.74 | 1.33 | 1.94 |
| **1041 s sputter time Intensity [CPS]** | 11939.62 | 1722.79 | 1636.09 | 3557.06 | 20750.46 | 3598.27 | 2723.91 | 20918.32 | 18469.06 | 2930.10 |
| **2002 s sputter time[%]** | 10.33 | 4.61 | 1.67 | 1.53 | 21.72 | 2.53 | 2.59 | 5.39 | 4.50 | 45.12 |
| **2002 s sputter time BE [eV]** | 284.80 | 286.06 | 288.55 | 290.10 | 283.85 | 399.19 | 397.88 | 531.72 | 528.75 | 55.10 |
| **2002 s sputter time FWHM [eV]** | 1.53 | 1.60 | 1.60 | 1.60 | 1.73 | 1.90 | 2.12 | 1.78 | 1.37 | 2.12 |
| **2002 s sputter time Intensity [CPS]** | 12052.24 | 5138.61 | 1859.59 | 1699.98 | 22442.12 | 3673.01 | 3387.00 | 13033.90 | 14246.68 | 2650.13 |

**
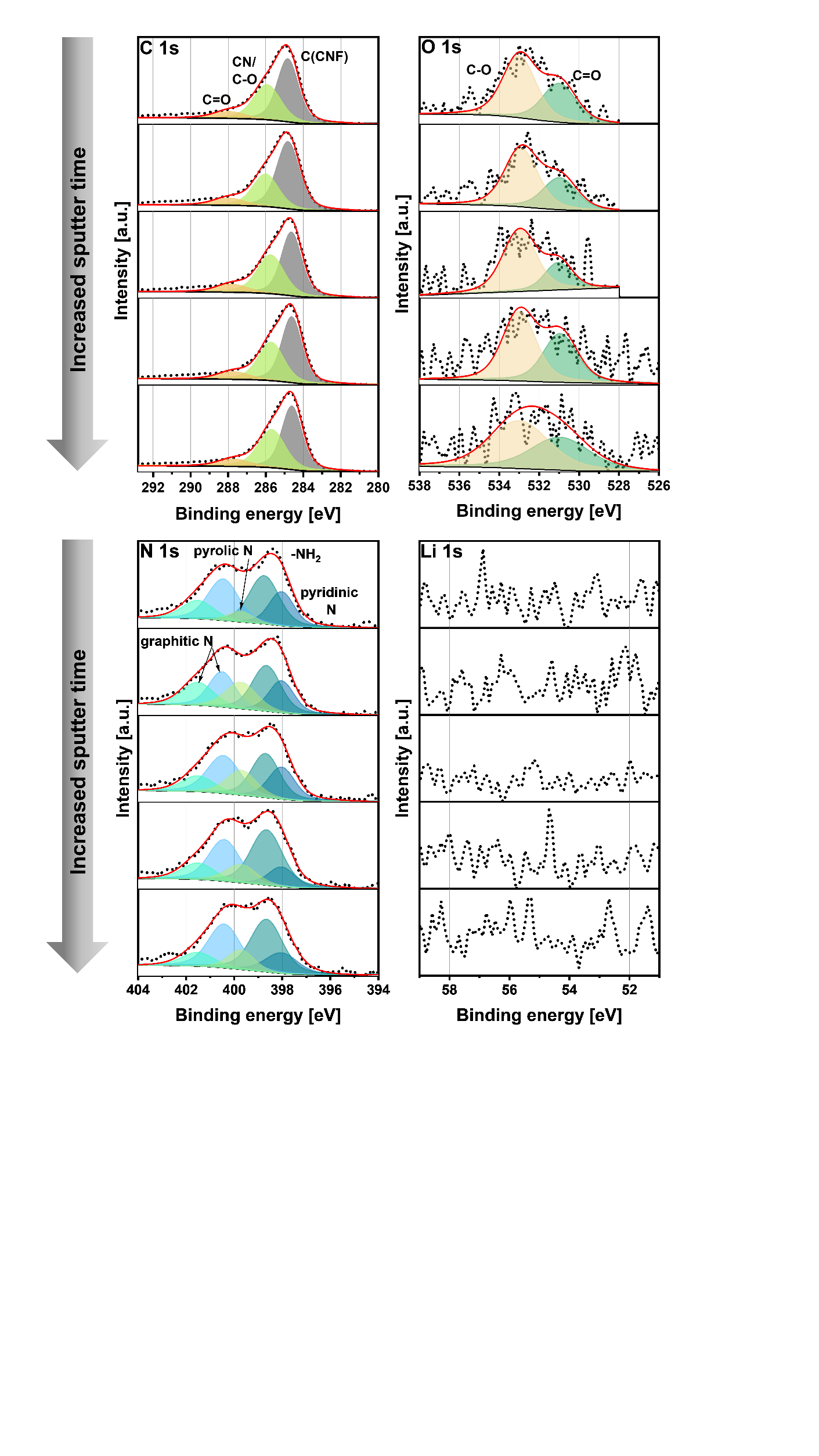
**

**Figure S4** C 1s, N 1s, O 1s, and Li 1s XPS spectra of CNF on the surface and after 10, 500, 1041, and 2002 s sputter time. The spectra for CNF are referenced to pyridinic N at 398.0 eV.  All spectra are normalized, with the highest signal in each spectrum set to 1. Measurement parameters can be found in **Table S2**. Fitting parameters can be found in **Table S3**.


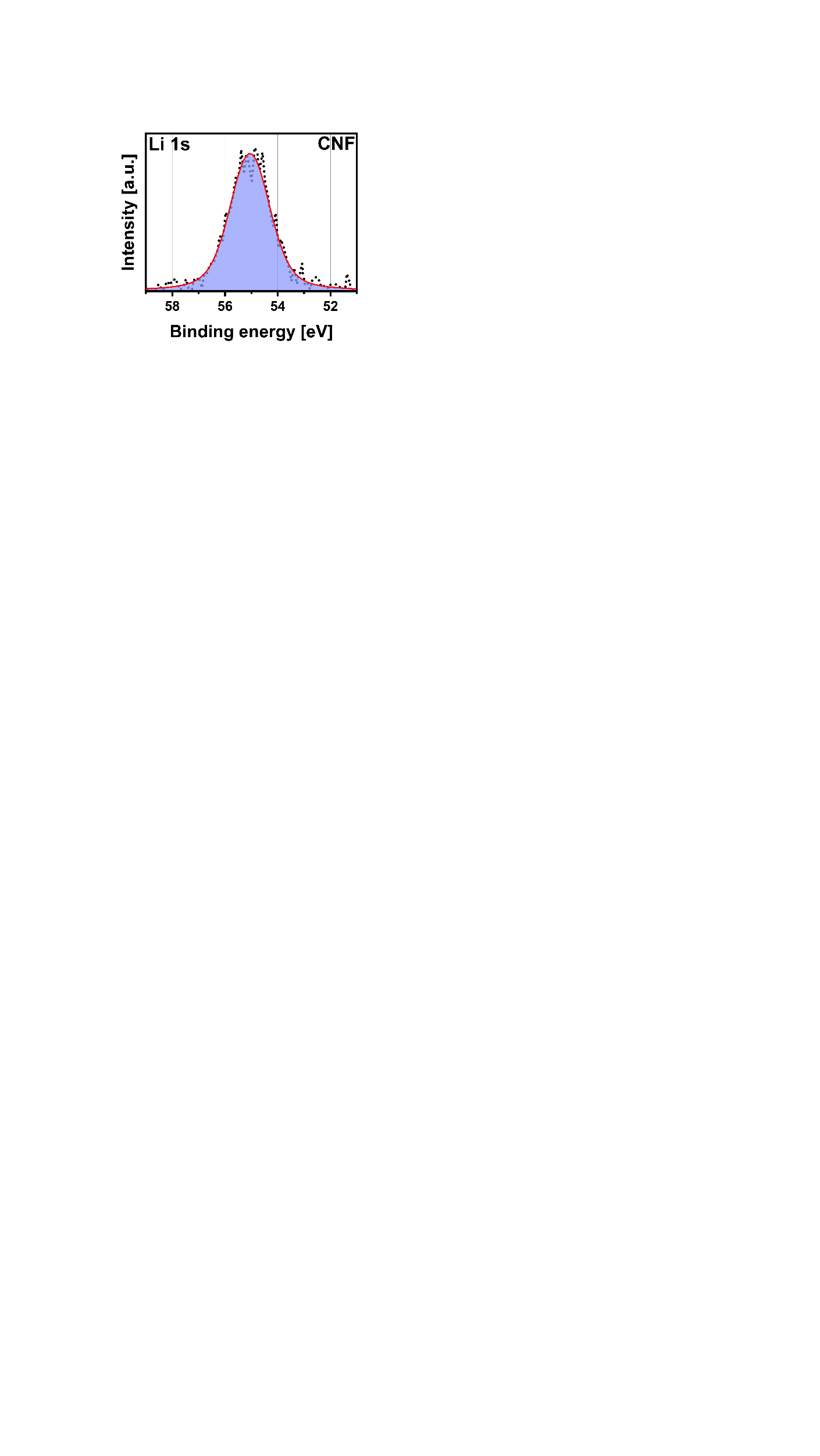


**Figure S5** Li 1s spectrum of CNF_Prelith_. The spectrum is referenced to C-C/C-H at 284.8 eV.  All spectra are normalized, with the highest signal in each spectrum set to 1. Measurement parameters can be found in **Table S2**. Fitting parameters can be found in **Table S4**.


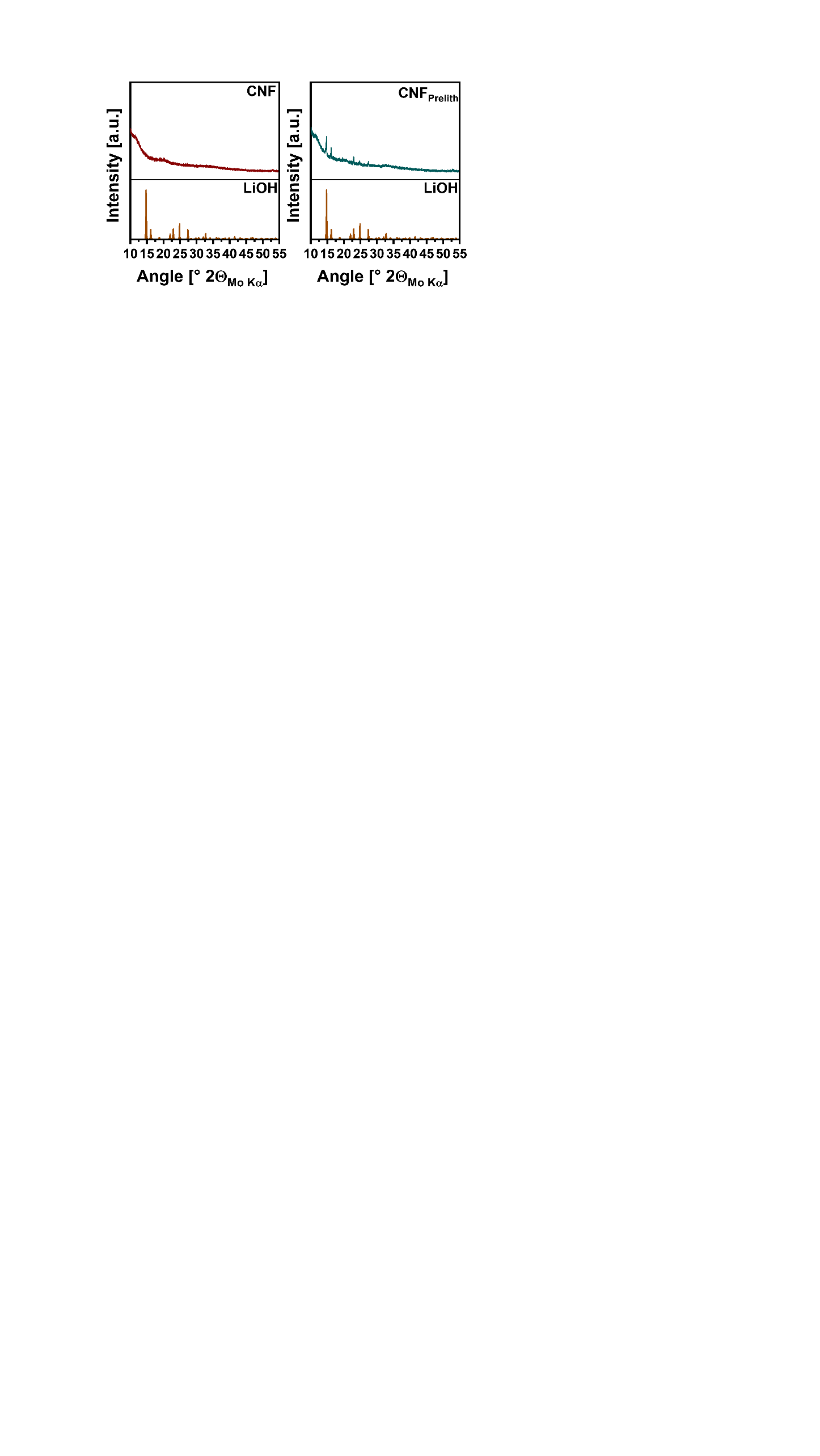


**Figure S6** XRD pattern of CNF and CNF_Prelith_ measured with Mo-Kα radiation in transmission mode. The reference XRD pattern is LiOH (ICSD 26892).


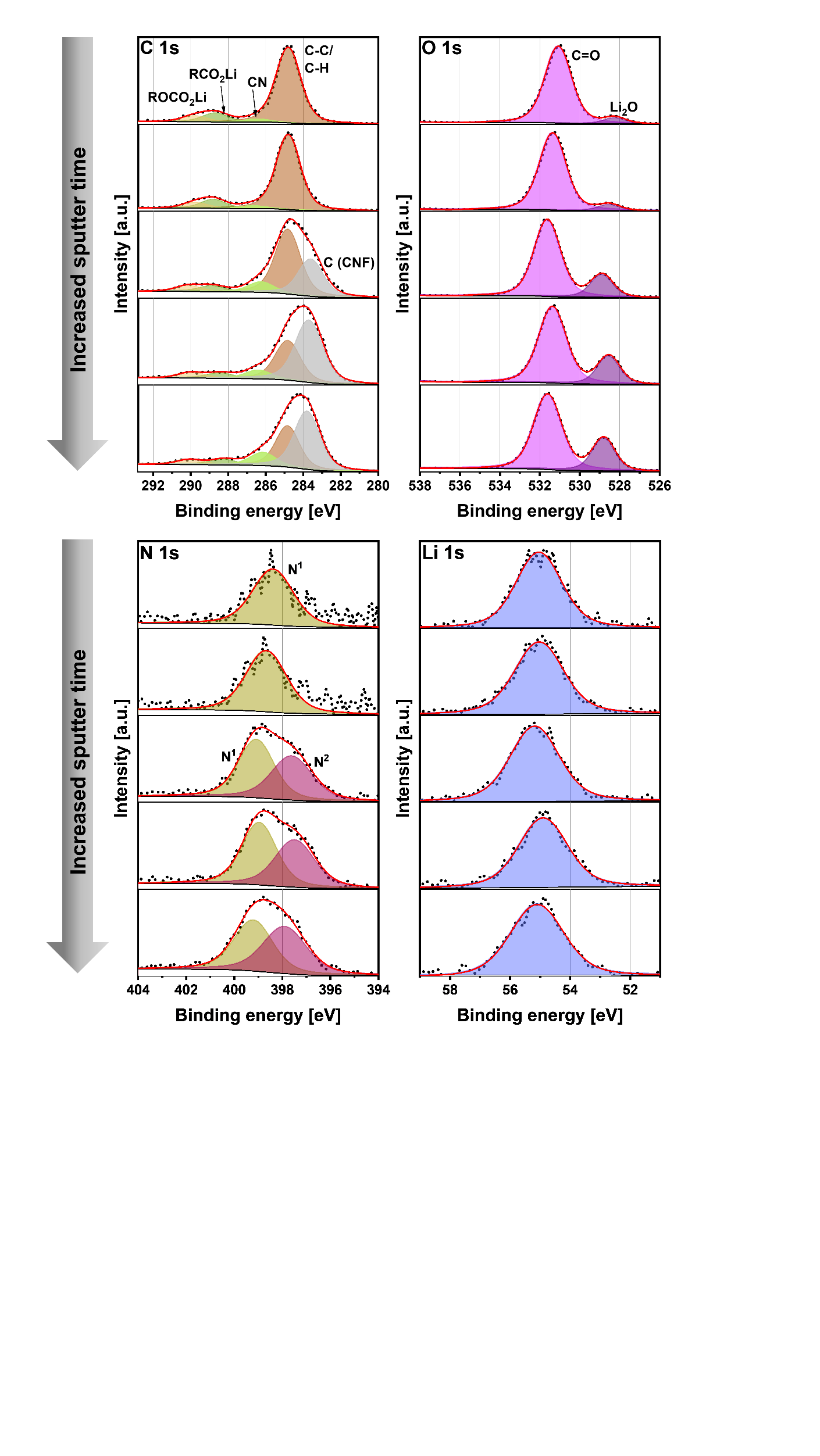


**Figure S7** C 1s, N 1s, O 1s, and Li 1s XPS spectra of CNF_Prelith_ on the surface and after 10, 500, 1041, and 2002 s sputter time. The spectra for CNF_Prelith_ are referenced to C-C/C-H at 284.8 eV.  All spectra are normalized, with the highest signal in each spectrum set to 1. Measurement parameters can be found in **Table S2**. Fitting parameters can be found in **Table S4**.


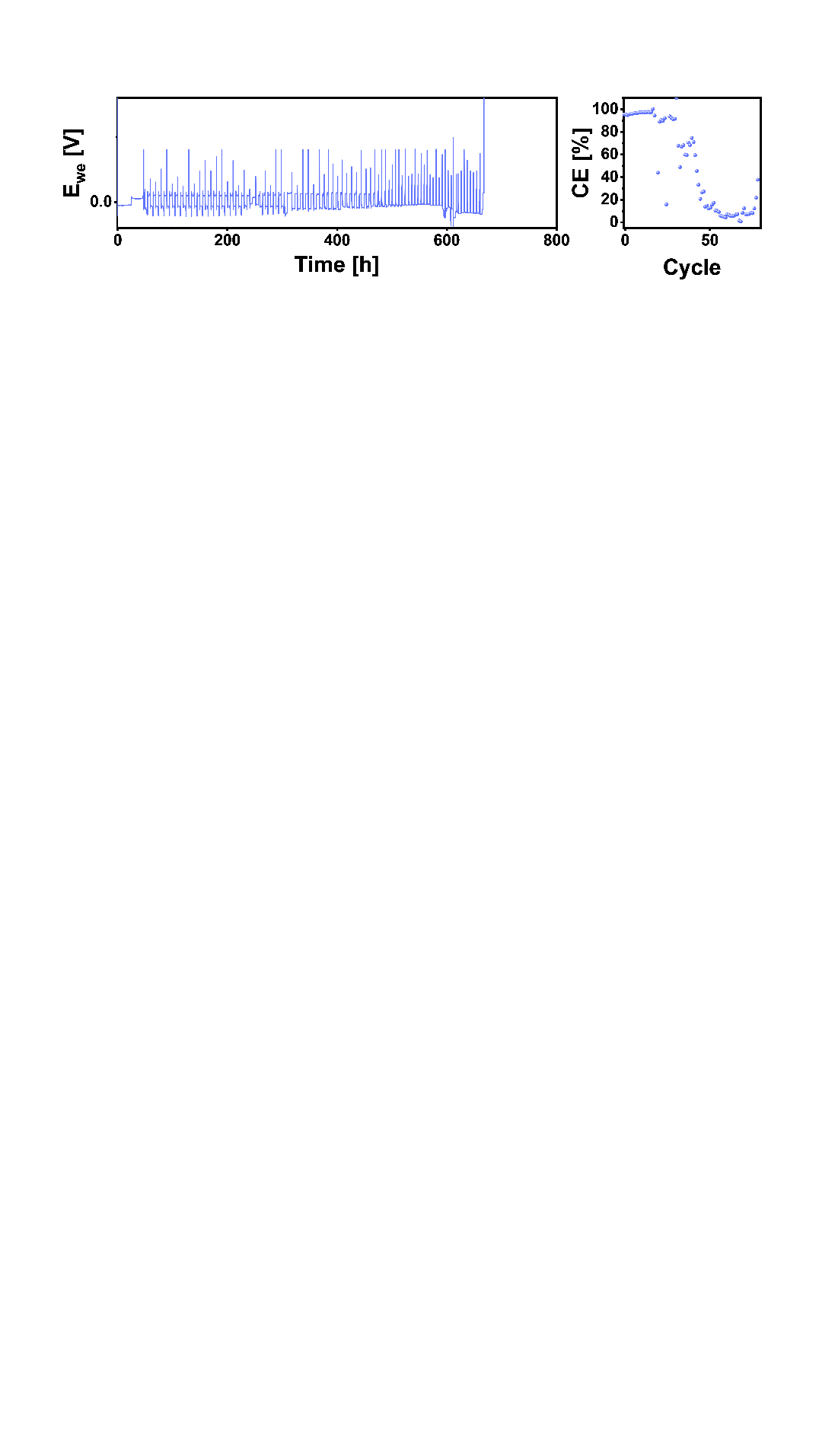


**Figure S8** Voltage - time profiles of bare Cu current collector during Li deposition/dissolution experiments at an areal capacity of 2.5 mA h cm^-2^. And the corresponding Coulombic efficiency of the cell.


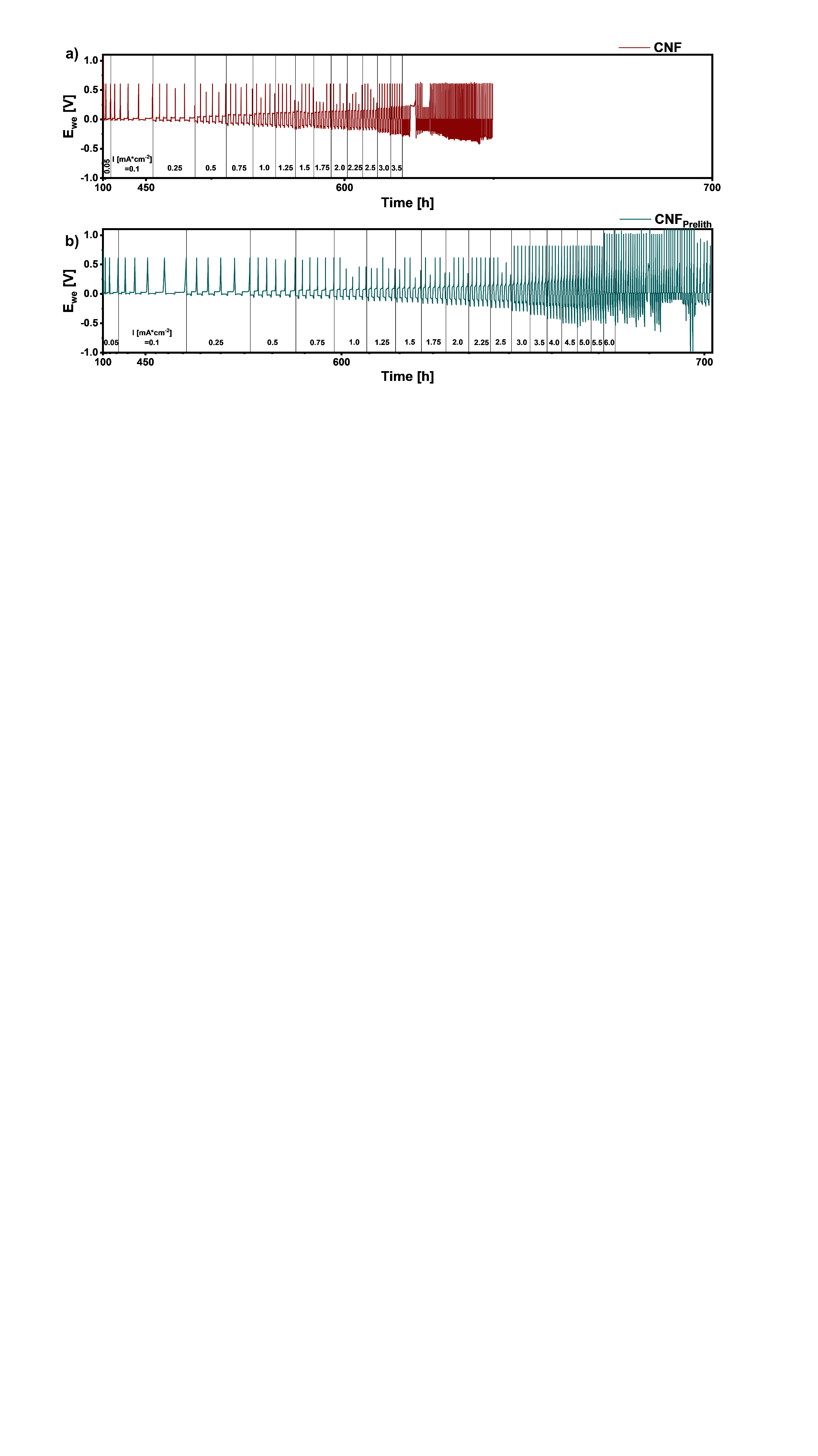


**Figure S9** Critical current test of CNF and CNF_Prelith._ The samples were tested in a Cu foil/sample||electrolyte||Li cell setup at room temperature. The cell capacity was fixed at 1.25 mA cm^-2^. After two formation steps at 0.05 mA cm^-2^_,_ the current was stepwise increased every 5 cycles.


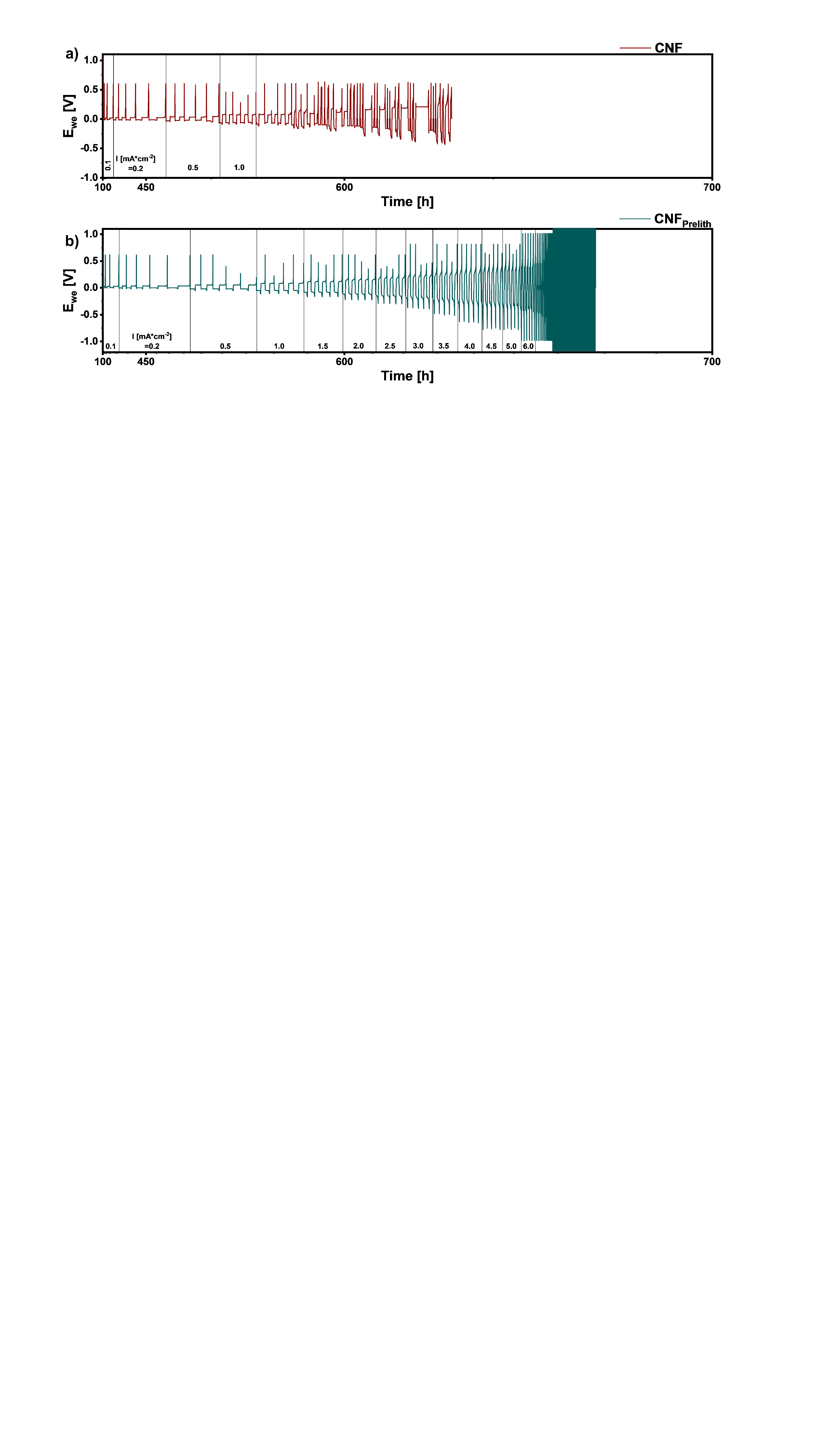


**Figure S10** Critical current test of CNF and CNF_Prelith._ The samples were tested in a Cu foil/sample||electrolyte||Li cell setup at room temperature. The cell capacity was fixed at 2.5 mA cm^-2^. After two formation steps at 0.1 mA cm^-2^_,_ the current was stepwise increased every 5 cycles.


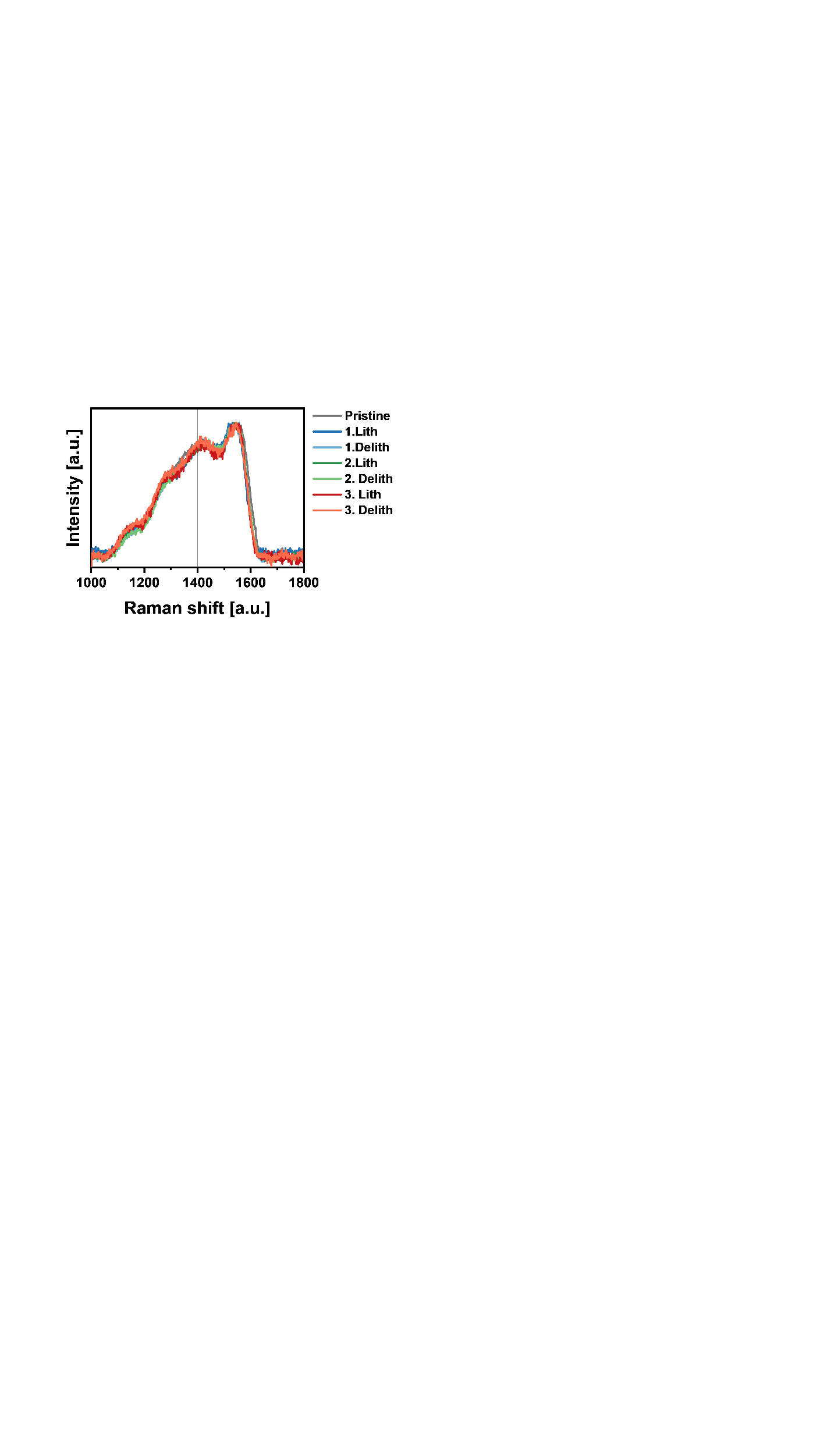


**Figure S11** Raman spectra of CNF_Prelith_ from the in-situ measurements at the end of each lithiation and delithiation half cycle.


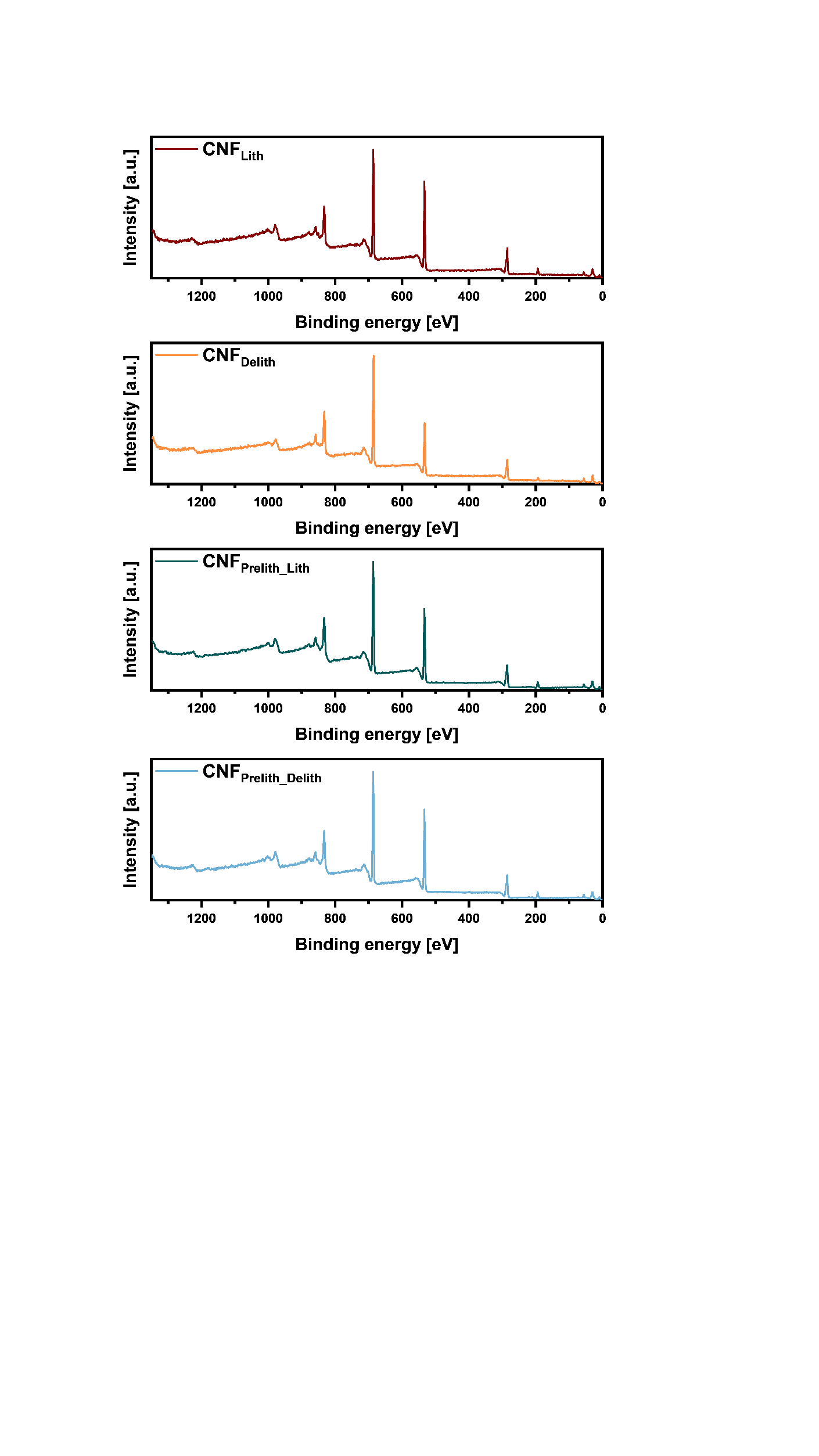


**Figure S12** Survey measurement of the XPS spectrum for CNF and CNF_Prelith_ in the lithiated and delithiated states. All spectra are referenced to C-C/C-H at 284.8 eV. All spectra are normalized, with the highest signal in each spectrum set to 1.

**Table S5** Quantification results and fitting parameters of cycled samples of CNF and CNF_Prelith_. Quantification refers to elements highlighted in bold. The BEs are referenced to C-C/C-H at 284.8 eV.

| **Component** | **C** (C-C/C-H)) | **C**-O | **C**=O | R**C**O_2_Li | RO**C**O_2_L**i** | **C**(CNF) | Li**F** | Li_x_B**F**_y_ | Li_x_B**F**_y_O_z_ | C-**O** | C=**O** | Li**O**H | Li_x_**B**F_y_ | Li_x_**B**F_y_O_z_ | Li**B**O_2_ | **N**^1^ | **N**^2^ | **N**^3^ | **Li** |
| --- | --- | --- | --- | --- | --- | --- | --- | --- | --- | --- | --- | --- | --- | --- | --- | --- | --- | --- | --- |
| **CNF Lith [%]** | 8.57 | 4.63 | 0.87 | 2.77 | 2.24 | 0.09 | 13.14 | 3.84 | 4.77 | 7.28 | 11.00 | 2.30 | 5.55 | 3.68 | 0.38 | 0.10 | 0.13 |  | 28.66 |
| **CNF Lith BE [eV]** | 284.80 | 286.42 | 288.00 | 289.10 | 290.05 | 283.05 | 685.25 | 687.33 | 686.51 | 533.44 | 532.32 | 530.70 | 192.91 | 194.40 | 190.80 | 396.27 | 400.60 |  | 55.95 |
| **CNF Lith FWHM [eV]** | 1.44 | 1.70 | 1.56 | 1.70 | 1.64 | 1.70 | 1.82 | 1.64 | 1.40 | 1.75 | 1.72 | 2.20 | 1.90 | 2.00 | 1.22 | 1.60 | 2.00 |  | 2.44 |
| **CNF Lith Intensity [CPS]** | 13858.08 | 6344.67 | 1290.97 | 3786.93 | 3178.44 | 126.46 | 51013.42 | 16537.02 | 24078.36 | 23419.74 | 36208.47 | 5907.31 | 2716.68 | 1707.59 | 292.40 | 216.85 | 233.39 |  | 1915.78 |
|  |  |  |  |  |  |  |  |  |  |  |  |  |  |  |  |  |  |  |  |
| **CNF Delith [%]** | 7.93 | 4.53 | 1.45 | 1.99 | 1.88 | 0.71 | 18.03 | 3.84 | 2.47 | 6.04 | 6.86 | 3.63 | 2.47 | 1.57 | 1.22 | 0.17 | 0.11 |  | 34.73 |
| **CNF Delith BE [eV]** | 284.80 | 286.31 | 287.91 | 288.92 | 290.06 | 282.96 | 685.16 | 687.24 | 686.42 | 533.40 | 532.05 | 530.63 | 192.71 | 194.69 | 191.10 | 396.42 | 399.43 |  | 55.89 |
| **CNF Delith FWHM[eV]** | 1.47 | 1.70 | 1.64 | 1.50 | 1.80 | 1.70 | 1.70 | 1.97 | 1.40 | 2.20 | 1.78 | 2.20 | 2.00 | 2.00 | 2.00 | 1.60 | 2.00 |  | 2.72 |
| **CNF Delith Intensity [CPS]** | 11619.64 | 5736.86 | 1898.62 | 2865.30 | 2240.69 | 902.77 | 69465.86 | 12682.06 | 11507.96 | 14276.72 | 20133.30 | 8604.98 | 1218.32 | 671.53 | 523.89 | 354.62 | 183.60 |  | 1918.60 |
|  |  |  |  |  |  |  |  |  |  |  |  |  |  |  |  |  |  |  |  |
| **CNF_Prelith_ Lith [%]** | 7.57 | 4.32 | 1.34 | 2.00 | 2.44 | 0.33 | 8.73 | 8.29 | 7.28 | 4.57 | 12.43 | 1.47 | 5.48 | 3.65 | 0.50 | 0.10 | 0.08 | 0.14 | 29.29 |
| **CNF_Prelith_ Lith BE [eV]** | 284.80 | 286.35 | 287.91 | 289.01 | 290.01 | 282.96 | 685.27 | 687.30 | 686.39 | 533.70 | 532.51 | 530.57 | 193.25 | 194.69 | 191.04 | 396.20 | 399.39 | 401.08 | 55.95 |
| **CNF_Prelith_ Lith FWHM [eV]** | 1.55 | 1.70 | 1.68 | 1.49 | 1.70 | 1.70 | 1.66 | 1.97 | 1.71 | 1.75 | 1.97 | 2.26 | 2.00 | 1.84 | 2.00 | 1.60 | 2.00 | 2.00 | 2.71 |
| **CNF_Prelith_ Lith Intensity [CPS]** | 11763.13 | 6102.79 | 1914.71 | 3207.80 | 3429.80 | 468.25 | 38350.68 | 30648.67 | 30891.37 | 15155.69 | 36544.25 | 3788.24 | 2620.36 | 1895.20 | 237.87 | 233.57 | 145.95 | 257.70 | 1811.98 |
|  |  |  |  |  |  |  |  |  |  |  |  |  |  |  |  |  |  |  |  |
| **CNF_Prelith_ Delith [%]** | 8.22 | 3.91 | 1.20 | 2.30 | 2.05 | 0.73 | 8.72 | 7.60 | 6.97 | 6.10 | 11.93 | 1.44 | 5.31 | 4.06 | 0.48 | 0.20 | 0.10 | 0.15 | 28.54 |
| **CNF_Prelith_ Delith BE[eV]** | 284.80 | 286.45 | 288.04 | 289.05 | 290.00 | 283.00 | 685.28 | 687.36 | 686.44 | 533.68 | 532.63 | 530.66 | 193.34 | 194.78 | 191.03 | 397.22 | 399.43 | 401.12 | 56.00 |
| **CNF_Prelith_ Delith FWHM[eV]** | 1.58 | 1.70 | 1.60 | 1.49 | 1.70 | 1.73 | 1.57 | 1.83 | 1.53 | 1.90 | 1.96 | 1.83 | 1.98 | 1.97 | 1.46 | 1.60 | 1.97 | 2.00 | 2.60 |
| **CNF_Prelith_ Delith Intensity [CPS]** | 10490.26 | 4636.58 | 1508.42 | 3093.54 | 2373.02 | 862.42 | 33980.28 | 25401.42 | 27803.22 | 15652.28 | 29606.73 | 3842.35 | 2149.23 | 1649.78 | 263.08 | 400.24 | 158.76 | 234.79 | 1544.28 |


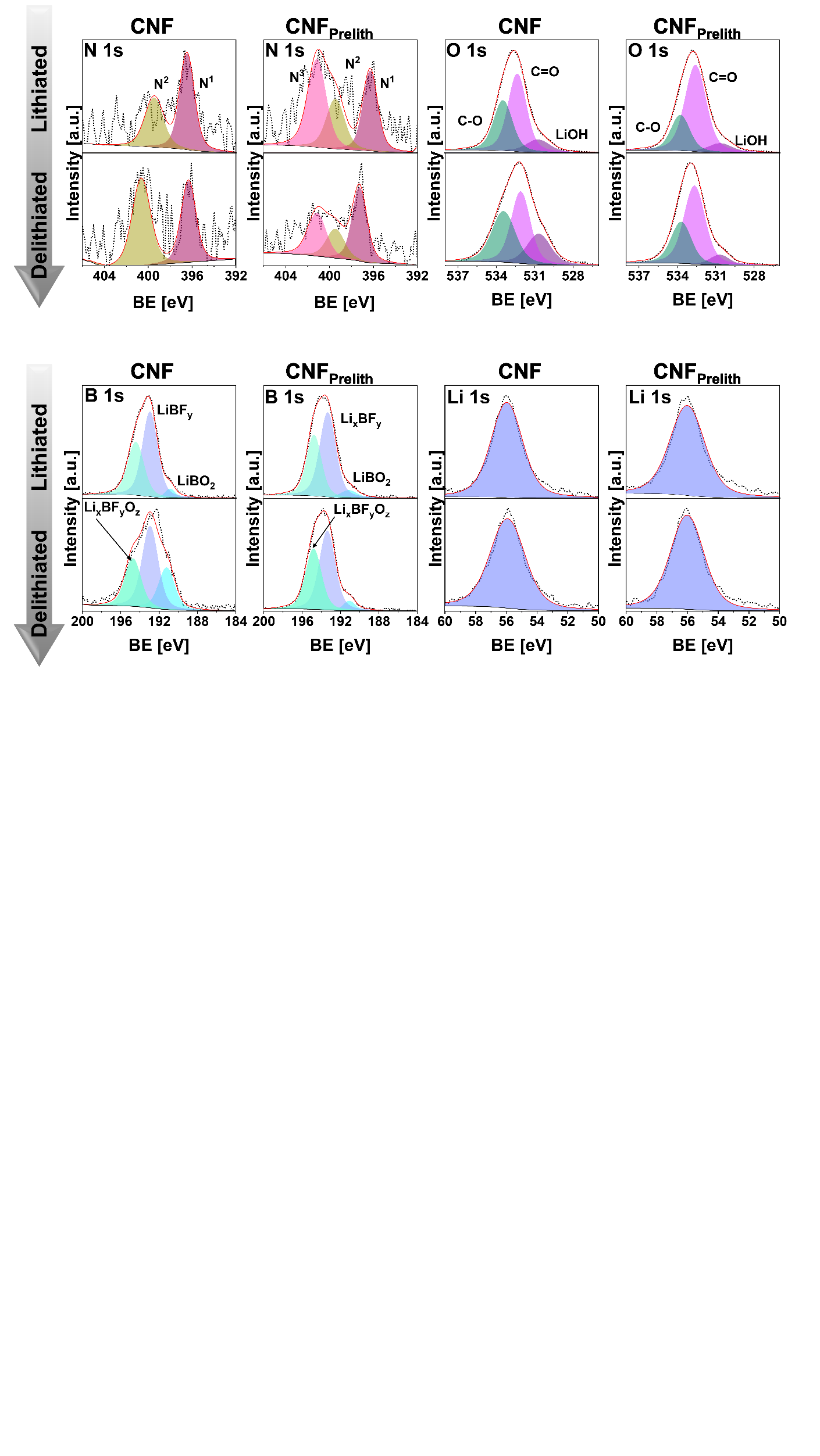


**Figure S13** N 1s, O 1s, B 1s, and Li 1s XPS spectra of CNF and CNF_Prelith_ in the lithiated and delithiated state. The spectra are referenced to 284.8 eV for the signal corresponding to C-C/C-H. All spectra are normalized, with the highest signal in each spectrum set to 1. Survey measurements can be found in **Figure S10**. Detailed measurement parameters can be found in **Table S2**. Fitting parameters can be found in **Table S5**.


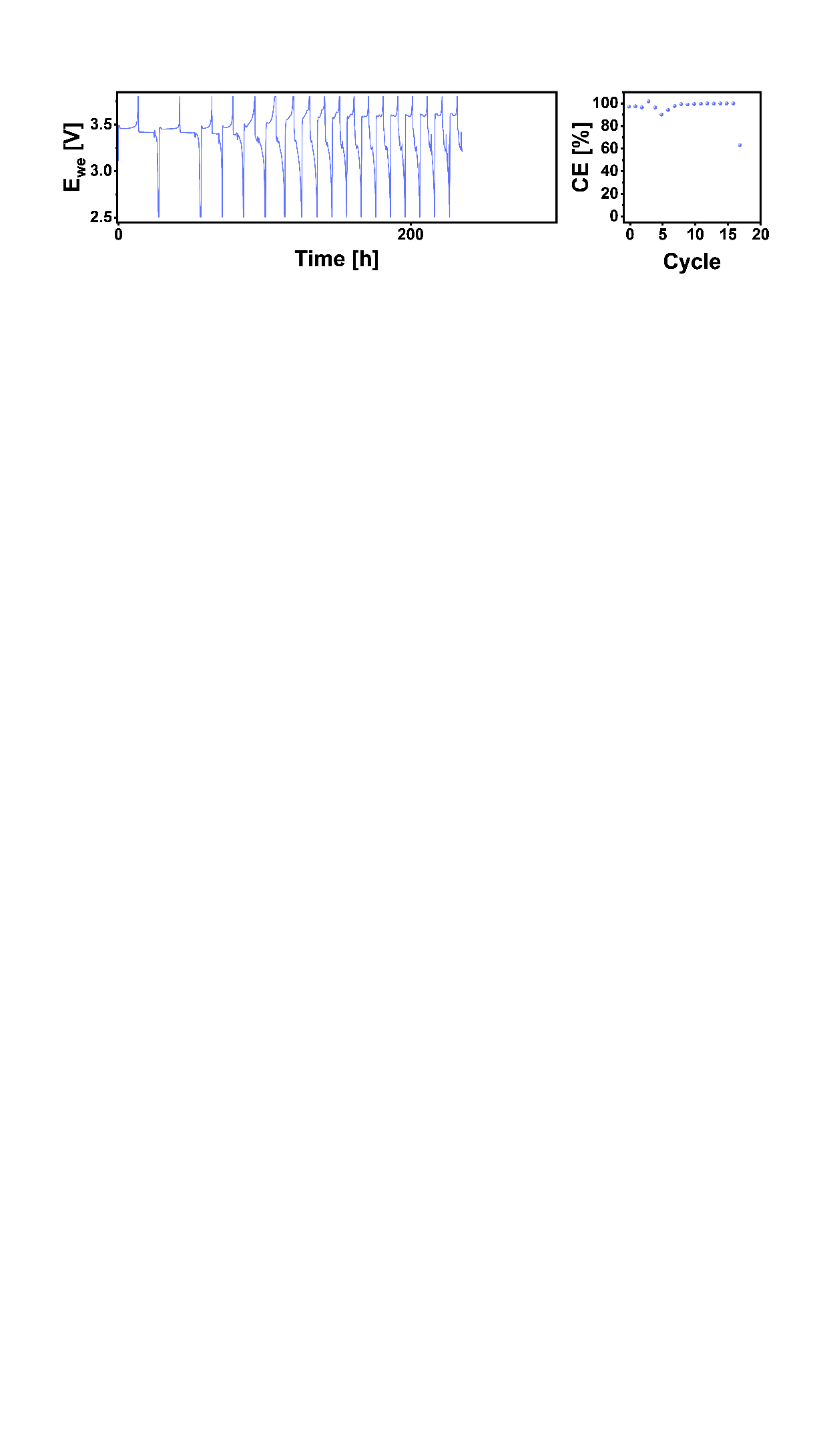


**Figure S14** Voltage - time profiles of bare Cu current collector under zero-excess lithium metal conditions vs. LFP. And the corresponding Coulombic efficiencies of the cell.

**Table S6** List of inversion recovery NMR delays.

| **Delay**  **[s]** |
| --- |
| 0.004 |
| 0.019 |
| 0.035 |
| 0.05 |
| 0.066 |
| 0.081 |
| 0.1875 |
| 0.375 |
| 0.6 |
| 1.35 |
| 6.943 |
| 12.536 |
| 18.129 |
| 23.721 |
| 29.314 |
| 61.714 |
| 111.429 |
| 161.143 |
| 210.857 |
| 260.571 |
| 600.0 |
| 2000 |


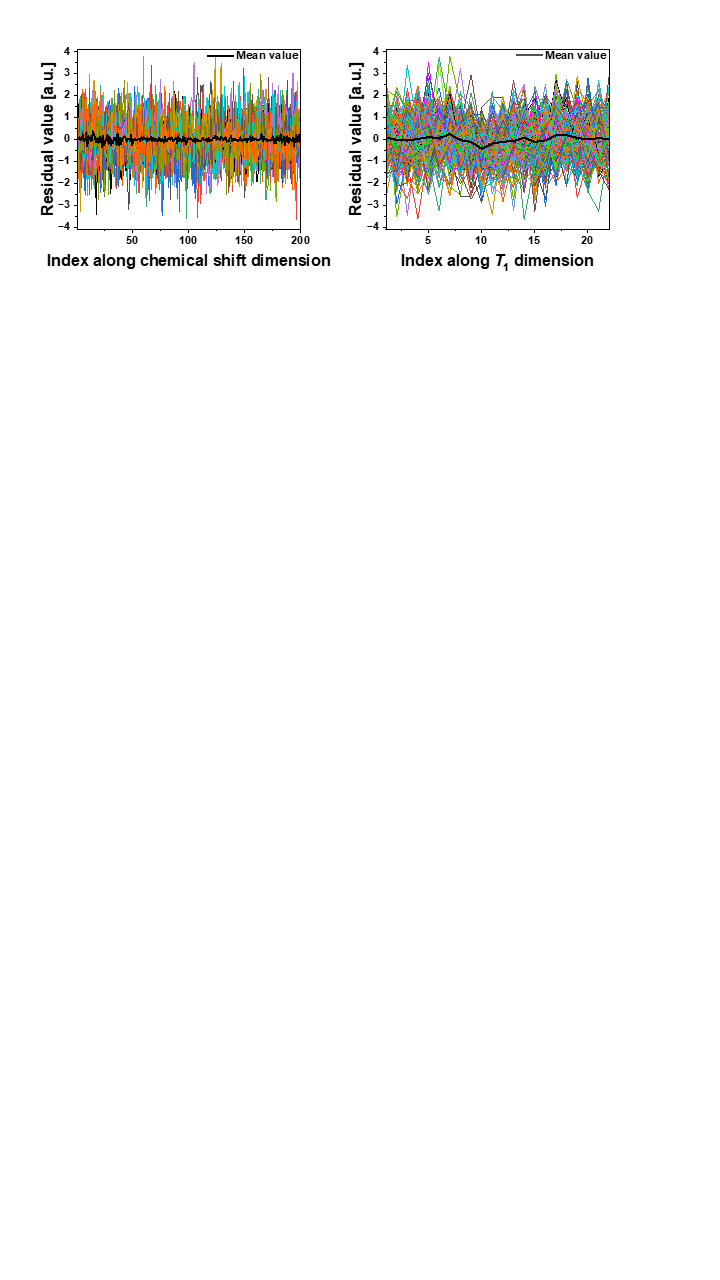


**Figure S15** The residual values obtained from the ILT are depicted for the index along the chemical shift dimension, as well as for the index along the T_1_ dimension.
